# A type III ACC synthase, *ACS7*, is involved in root gravitropism in *Arabidopsis thaliana*

**DOI:** 10.1093/jxb/ert241

**Published:** 2013-08-13

**Authors:** Shih-Jhe Huang, Chia-Lun Chang, Po-Hsun Wang, Min-Chieh Tsai, Pang-Hung Hsu, Ing-Feng Chang

**Affiliations:** ^1^Institute of Plant Biology, National Taiwan University, Taipei, Taiwan; ^2^Institute of Bioscience and Biotechnology, National Taiwan Ocean University, Keelung, Taiwan; ^3^Department of Life Science, National Taiwan University, Taipei, Taiwan

**Keywords:** 14-3-3, ACS, calcium, CDPK, ethylene, phosphorylation, root gravitropism.

## Abstract

Ethylene is an important plant hormone that regulates developmental processes in plants. The ethylene biosynthesis pathway is a highly regulated process at both the transcriptional and post-translational level. The transcriptional regulation of these ethylene biosynthesis genes is well known. However, post-translational modifications of the key ethylene biosynthesis enzyme 1-aminocyclopropane-1-carboxylate (ACC) synthase (ACS) are little understood. *In vitro* kinase assays were conducted on the type III ACS, AtACS7, fusion protein and peptides to determine whether the AtACS7 protein can be phosphorylated by calcium-dependent protein kinase (CDPK). AtACS7 was phosphorylated at Ser216, Thr296, and Ser299 by AtCDPK16 *in vitro*. To investigate further the function of the *ACS7* gene in *Arabidopsis*, an *acs7-1* loss-of-function mutant was isolated. The *acs7-1* mutant exhibited less sensitivity to the inhibition of root gravitropism by treatment with the calcium chelator ethylene glycol tetraacetic acid (EGTA). Seedlings were treated with gradient concentrations of ACC. The results showed that a certain concentration of ethylene enhanced the gravity response. Moreover, the *acs7-1* mutant was less sensitive to inhibition of the gravity response by treatment with the auxin polar transport inhibitor 1-naphthylphthalamic acid, but exogenous ACC application recovered root gravitropism. Altogether, the results indicate that *AtACS7* is involved in root gravitropism in a calcium-dependent manner in *Arabidopsis*.

## Introduction

Gravity is one of the most important environmental cues that control growth direction (Morita, 2010). Shoots generally grow upward (i.e. negative gravitropism) and roots grow downward (i.e. positive gravitropism). It is unknown how plants receive and respond to the gravity signal. One widely accepted theory is that starch-accumulating amyloplast movement along the gravity vector within gravity-sensing cells (statocytes) is a likely trigger of subsequent intracellular signalling (Morita, 2010). However, several studies have demonstrated that starch is important but not essential for gravity sensing (Strohm *et al.*, 2011; Wolverton *et al.*, 2011). The receptor that responds to gravity has not yet been identified. In the process of gravity perception, changes in the gravity vector are transduced into multiple intracellular signals [i.e. cytosolic pH, inositol 1,4,5-triphosphate (InsP_3_), and cytosolic calcium concentration] (Fasano *et al.*, 2001; Plieth *et al.*, 2002; Perera *et al.*, 2006). The plant hormone auxin was identified as one of these signalling molecules, and its redistribution is polar. Auxin has been thought to be involved in gravitropic responses, based on the Cholodny–Went theory. Auxins were the first group of plant growth regulatory substances to be discovered (Went, 1928). Indole-3-acetic acid (IAA) was the first native representative of the group that was identified. Auxins are known to be involved in the regulation of basic growth processes, such as cell division and cell elongation at the tissue, organ, and whole-plant levels. The auxin molecule functions as a mobile signal between cells, tissues, and organs, and is involved in the spatial and temporal coordination of plant morphogenesis and responses to the environment *in planta*. Auxin is not only a mobile molecule; it also participates in downstream signalling. Many developmental processes appear to depend on the local asymmetric distribution of auxin molecules (Tanaka *et al.*, 2006). These include embryo development and apical–basal axis formation in *Arabidopsis thaliana* (Friml *et al.*, 2003), pattern formation and root development (Blilou *et al.*, 2005), organ formation (Benkova *et al.*, 2003), and phototropism and gravitropism (Blakeslee *et al.*, 2004).

In addition to auxin, the phytohormone ethylene has been shown to be involved in the gravity response in plants (Madlung *et al.*, 1999). In tomatoes, root penetration into the soil required cross-talk between ethylene and auxin (Santisree *et al.*, 2011). A mutation of the *Arabidopsis* gene *ARG1*, which is involved in root gravitropism, showed resistance to ethylene but increased sensitivity to auxin (Sedbrook *et al.*, 1998). The *AGR* gene encodes a membrane protein that is homologous with bacterial transporter proteins (Utsuno *et al.*, 1998) as a polar auxin transporter (Chen *et al.*, 1998). Another *Arabidopsis* mutant, *clg1*, showed resistance to ethylene in root gravitropism (Ferrari *et al.*, 2000). In *Arabidopsis*, flavonoid accumulation by ethylene was found to be involved in root gravitropism (Buer *et al.*, 2006). Additionally, the *Arabidopsis* mutant *rha1* showed resistance to ethylene in roots and was found to be involved in root gravitropism (Fortunati *et al.*, 2008). In six plant species tested, an ethylene biosynthesis inhibitor inhibited root curvature (Hoson *et al.*, 1996). In *Arabidopsis*, a recent study showed that ethylene and gravity can affect root skewing and waving (Oliva and Dunand, 2007). The *Arabidopsis* mutant *alh1* was shown to link the cross-talk between ethylene and auxin in the gravity response in roots (Vandenbussche *et al.*, 2003). Moreover, *acs6* and *acs9* mutants showed defective hypocotyl length in the gravity response (Tsuchisaka *et al.*, 2009). However, it is still controversial whether ethylene plays a positive or negative role in modulating the gravity response (Philosoph-Hadas *et al.*, 1996; Buer *et al.*, 2006).

Ethylene is a plant hormone involved in many plant growth and developmental processes, including seed germination, leaf and flower senescence and abscission, cell elongation, fruit ripening, nodulation, and responses to a wide variety of stressors (Crocker and Knight, 1908; Wang *et al.*, 2002; Yoo *et al.*, 2009). The biosynthesis of ethylene has been well documented in plants (Yang and Hoffman, 1984; McClellan and Chang, 2008). Ethylene is derived from the amino acid methionine, which is converted to *S*-adenosylmethionine (AdoMet) by *S*-adenosylmethionine synthetase. AdoMet is then converted to 1-aminocyclopropane-1-carboxylic acid (ACC) and 5′-deoxy-5′methylthioadenosine (MTA) by the enzyme 1-aminocyclopropane-1-carboxylase synthase (ACS) (Adams and Yang, 1979; Lin *et al.*, 2009), which is the rate-limiting step in ethylene biosynthesis. MTA is recycled to methionine through the Yang cycle, which allows high rates of ethylene production without depleting endogenous methionine. ACC is converted to ethylene, CO_2_, and cyanide by ACC oxidase (ACO). The cyanide produced by this reaction is detoxified into β-cyanoalanine by the enzyme β-cyanoalanine synthase, preventing toxicity to plants under conditions of high ethylene biosynthesis.

ACS proteins play an important role in the ethylene biosynthesis pathway. The enzyme catalyses the conversion of AdoMet to ACC, and this reaction requires pyridoxal-5′-phosphate (PLP) as a cofactor. In most plant species, ACS is encoded by a multigene family that is regulated by various environmental and developmental factors (i.e. cytokinin, auxin, root hair development, fruit ripening, wounding, and pathogens). *Arabidopsis* has eight genes that encode active ACS proteins and an additional gene that encodes a catalytically inactive enzyme, ACS1 (Liang *et al.*, 1992). The *Arabidopsis ACS2*, *ACS6*, *ACS7*, and *ACS9* genes can be induced by hypoxia (Peng *et al.*, 2005). Zhang’s group found that *ACS2*, *ACS6*, *ACS7*, *ACS8*, and *ACS11* were involved in *Botrytis cinerea*-induced ethylene biosynthesis in *Arabidopsis* (Li *et al.*, 2012). Based on the C-terminal sequences, ACS proteins in *Arabidopsis* can be divided into three main types (Chae and Kieber, 2005). Type I proteins have an extended C-terminus that contains three conserved serine residues that are targets for phosphorylation by mitogen-activated protein kinase 6 (MPK6) (Liu and Zhang, 2004) and a conserved serine residue that is a phosphorylation site for calcium-dependent protein kinase (CDPK; Tatsuki and Mori, 2001; Sebastià *et al.*, 2004). Type II proteins have a shorter C-terminus that has only the CDPK phosphorylation site. Type III proteins have a very short C-terminal extension that lacks both phosphorylation sites. ACS proteins can act as homo- or heterodimeric proteins, similar to other PLP-dependent enzymes, and their ability to form active heterodimers might act to increase the versatility of ethylene responses (Tsuchisaka and Theologis, 2004), which enhances the ability to regulate ethylene production after exposure to different developmental and environmental stimuli.

Details on ACS turnover have been derived from studies of *Arabidopsis* ethylene-overproducing (Eto) mutants (Chae and Kieber, 2005). The *eto* mutants produce 10- to 40-fold more ethylene in the dark compared with the wild-type (WT) seedlings, and adopt a triple-response morphology (i.e. a morphology that etiolated seedlings adopt in the presence of ethylene) in the absence of exogenous ethylene application (Guzman and Ecker, 1990; Kieber *et al.*, 1993). The cloning of *ETO1* revealed that it encodes an E3 ligase component, a BTB/TPR protein. ETO1 binds to type II ACS proteins but not type I or type III ACS proteins (Wang *et al.*, 2004; Yoshida *et al.*, 2005, 2006). The disruption of ETO1 resulted in increased stability of the type II ACS protein ACS5 (Chae *et al.*, 2003) and consequently increased ethylene biosynthesis. The stability of ACS proteins is also regulated by protein phosphorylation. Treatment of tomato cells with the protein kinase inhibitors K-252a and staurosporine inhibited the elicitor-dependent induction of ACS and ethylene biosynthesis (Grosskopf *et al.*, 1990; Felix *et al.*, 1991) through a mechanism that most probably involves increased turnover of the ACS protein (Spanu *et al.*, 1994). In tomato cells, ACS2 was shown to be phosphorylated by CDPK from extracts of wounded tomato fruit (Tatsuki and Mori, 2001). The protein stability of ACS2 was found to be regulated by CDPK phosphorylation (Kamiyoshihara *et al.*, 2010). The target of CDPK phosphorylation was the conserved serine residue Ser460 at the C-terminal region of the ACS protein. A novel CDPK phosphorylation motif was identified in the C-terminal domain of type II ACS proteins (Sebastià *et al.*, 2004). The current model proposes that the phosphorylation of type I and type II ACS proteins blocks the ability of ETO1/EOL proteins to bind and inhibit the ubiquitination of these ACS proteins for their degradation by the 26S proteasome (Wang *et al.*, 2004; Chae and Kieber, 2005).

The regulation of ethylene biosynthesis and ACS stability are also controlled by mitogen-activated protein kinases (MAPKs). In tobacco, a stress-induced MAPK (SIPK) is involved in the response to different stressors, including pathogen- and ozone-induced ethylene biosynthesis. The closest homologue of SIPK in *Arabidopsis* is MPK6. Therefore, MPK6 was used in an *in vitro* kinase assay. The results showed that MPK6 can phosphorylate ACS2 and ACS6 *in vitro*, and transgenic plants that overexpress a phosphomimic mutant of ACS6 showed increased ethylene production (Liu and Zhang, 2004). These results indicate that a pathway similar to the SIPK pathway in tobacco operates in *Arabidopsis* and that MPK6 phosphorylates ACS proteins, thereby decreasing their turnover and increasing ethylene biosynthesis after pathogen stress. A possible CDPK- and MPK6-regulated pathway was recently proposed by Ludwig *et al.* (2005). These findings highlight the complexity of phosphorylation-regulated signalling and ethylene biosynthesis in plants in response to different stressors.

Calcium is a ubiquitous secondary messenger in eukaryotic cells. In plants, intracellular calcium levels can modulate many growth and developmental processes, including plant hormones, light, gravity, and biotic and abiotic stress (Batistic and Kudla, 2012). Unlike most other ions, calcium does not freely diffuse within cells (Trewavas, 1999). Plants have multiple calcium stores, including in apoplasts, vacuoles, the nuclear envelope, the endoplasmic reticulum (ER), chloroplasts, and mitochondria. Different stimuli can trigger calcium efflux from specific organelles. After calcium is released, different calcium sensors that have an EF-hand motif that can specifically bind calcium [i.e. the EF-hand-containing proteins calmodulin (CaM) and CDPK] can recognize specific calcium signals in specific places and transduce them into downstream effects, including altered protein phosphorylation and gene expression patterns (Sanders *et al.*, 1999, 2002; Dodd *et al.*, 2010; Perochon *et al.*, 2011; Liese and Romeis, 2012).

In *Arabidopsis*, the *C*-terminal domain in type I and type II ACS proteins can be phosphorylated by CDPK and MAPK. This phosphorylation of type I and type II ACS proteins blocks the ability of ETO1/EOL proteins to bind, thus inhibiting the ubiquitination of these ACS proteins and their degradation by the 26S proteasome. The degradation of type III ACS7 was recently found to also be mediated by the 26S proteasome (Lyzenga *et al.*, 2012), but how ACS7 protein activity and stability are regulated is still unknown. The present study investigated whether the type III ACS protein ACS7 is phosphorylated by CDPK *in vitro*. An *in vitro* kinase assay was conducted to determine the phosphorylation of ACS7 by CDPK and identify the phosphorylation site in ACS7. Additionally, the protein–protein interaction between ACS7 and 14-3-3ω was confirmed using two independent methods. A T-DNA insertion knockout mutant, *acs7-1*, was also identified and used to investigate the previously uncharacterized functions of *ACS7* in *Arabidopsis*. It was found that *ACS7* is involved in root gravitropism in *Arabidopsis*.

## Materials and methods

### Plant growth conditions

Seeds from WT *Arabidopsis thaliana* plants (Columbia and Wassilewskija ecotype) were sterilized with chlorine for 3h with 100ml of 6% bleach and 3ml of 10 N sulphuric acid (H_2_SO_4_) and then spread onto plates that contained half-strength Murashige and Skoog medium and 0.5% sucrose. The seeds were placed in the dark for 2 d at 4 °C and then incubated in growth chambers for 16h with 100 μmol photon m^–2^ s^–1^ light and 8h in the dark at 23 °C. After seed germination, all of the plants were transferred to a 9F walk-in growth chamber under a short-day photoperiod condition (8h light/16h dark) or a long-day photoperiod condition (16h light/8h dark).

### Preparation of E. coli DH5α competent cells for transformation

*Escherichia coli* competent cells were prepared. A single colony of DH5α was inoculated into 5ml of LB medium (1% tryptone, 0.5% yeast extract, and 1% NaCl) with shaking at 37 °C for 16h. The cells were then added to a 1 litre flask that contained 500ml of Super Optimal Broth medium with shaking at 37 °C for 3h. The cells were centrifuged at 2700 *g* (Beckman Coulter J2-MC, Brea, CA, USA) for 10min at 4 °C and gently resuspended in 130ml of 0.1M CaCl_2_ solution. The cells were then centrifuged at 2700 *g* for 10min at 4 °C, and the supernatant was removed. Finally, the cells were resuspended in 4ml of TB buffer and separated in a 1.5ml tube. DH5α competent cells were placed on ice, and 1 μl of plasmid was added to thaw the cells. The cells were placed on ice for 30min and heat-shocked for 90 s at 42 °C. The cells were then placed on ice for 2min. LB medium (1ml) was added to let the cells recover at 37 °C for 30min. After recovery, the cells were spread on plates and incubated overnight at 37 °C.

### Purification of glutathione S-transferase (GST)-tagged protein

*Escherichia coli* was incubated in 40ml of 2× YT medium with 200 μg of ampicillin at 37 °C for 16–18h overnight, and 400ml of 2× YT medium was then added at 28 °C for 3h. After incubation, 220 μl of isopropyl β-d-1-thiogalactopyranoside (IPTG; 1M stock, 230mg ml^–1^) was added to a final concentration of 0.5mM and incubated for 3h at 37 °C. The cells were centrifuged at 6000rpm (Beckman Coulter J2-MC) for 30min at 4 °C. The supernatant was discarded, and 20ml of lysis buffer was then added for 15min. The cells were transferred to a 50ml Falcon tube and stored at –80 °C. The cells were incubated in water (~40 °C), and a sonicator (Misonix XL2020, Farmingdale, NY, USA) was used to break the cells. After sonication, the cells lysate was centrifuged at 10 000rpm for 30min at 4 °C. The supernatant was then transferred to a new 50ml Falcon tube. GST beads (1ml) were added and washed with GST binding buffer three times, and the solution was shaken for 1–3h in a cold room. The GST beads were centrifuged, and 10ml of GST binding buffer was added to wash the beads three times. TRIS buffer (10ml, 50mM, pH 7.5) was added to wash the beads, and the solution was transferred to a biospin column (Bio-Rad, Hercules, CA, USA) to allow the GST beads to adhere to the column. TRIS buffer (1.5ml, 50mM, pH 8.0) that contained 10mM glutathione (30mg per 10ml) was used to elute the protein, and the eluate was collected by a centrifugal filter (Amicon Ultra 10K, Millipore, Billerica, MA, USA).

### 6His-SUMO–ACS7 recombinant protein purification

6His-SUMO–ACS7 recombinant protein was incubated in 40ml of LB medium that contained 50 μg ml^–1^ ampicillin and shaken at 37 °C overnight. The overnight culture was inoculated with 400ml of LB medium with shaking at 37 °C. The cells were raised to an absorbance of optical density (OD) 0.4–0.6 (mid-log phase) at 600nm and then induced with 0.5mM IPTG and allowed to continue to grow for 3h. The cells were then harvested by centrifugation at 5520 *g* (Beckman Coulter J2-MC) for 30min. The cell pellet was resuspended in lysis buffer and stored at –80 °C. The cells were thawed in 40 °C water, sonicated, and centrifuged at 20 400 *g* for 30min (Beckman Coulter J2-MC). The supernatant was incubated with pre-washed Ni-NTA resin (GE) for 2h at 4 °C and washed with a first wash buffer and second wash buffer. 6His protein was eluted with 1.5ml of elution buffer. The eluate was collected with a centrifugal filter (Amicon Ultra 10K, Millipore) for buffer exchange. The fusion protein was resuspended in phosphate-buffered saline (PBS) buffer. Plasmid maps are shown in Supplementary Fig. S1 available at *JXB* online.

### Protein quantification using the Bradford assay

The protein concentration measurement was based on the Bradford method using Protein Assay Dye (Bradford, 1976; catalog no. 500-0006, Bio-Rad). Protein assay dye (100 μl) was mixed with 900 μl of dH_2_O and added to different concentrations of bovine serum albumin to reconstitute the standard curve. Sample absorbance was read at 595nm.

### Fusion peptide design and construction

Approximately 50–60 nucleotide long forward and reverse primers were used to self-ligate in a temperature gradient and had a sticky-end *Asc*I and *Bam*HI restriction enzyme recognition site. After phosphorylation by polynucleotide kinase at the 5′ end for 30min, the double-stranded primer was constructed into an NRV vector and transformed into BL21 for fusion protein expression.

### In vitro kinase assay

The *in vitro* kinase assay was performed according to a modified method described previously (Curran *et al.*, 2011). ATP (50 μM, spiked with 1.25 μCi of [γ-^32^P]ATP) was added to begin the kinase reaction at a final volume of 10 μl, which consisted of 300ng of purified CDPK, 3 μg of fusion protein substrate, and standard kinase reaction buffer. The reactions were incubated for 15min at room temperature and stopped by SDS sample buffer. All of the samples were loaded onto a 12% SDS–PAGE loading well for electrophoresis, and labelling signals were normalized to the amount of protein determined from Coomassie Brilliant Blue-stained gels after running SDS–PAGE.

### Protoplast isolation

*Arabidopsis* protoplasts were isolated according to a modified method described previously (Yoo *et al.*, 2007). The leaves from 4-week-old plants were excised and subjected to an enzyme solution for 2h at room temperature. The enzyme solution that contained protoplasts was filtered with a miracloth and centrifuged at 100 *g* (Kubota 2420, Japan) to pellet the protoplasts in a 15ml tube for 3min. The supernatant was removed, and the protoplasts were washed three times in W5 solution. The protoplasts were resuspended in Mmg solution with 2.5×10^5^ protoplasts in 1ml before polyethylene glycol (PEG)-mediated transformation.

### Plasmid construction and transformation for transient expression

The open reading frame (ORF) of AtACS7 was amplified using designated primers from cDNA. The amplified ORF was inserted into the p2YGW7 vector (Invitrogen). The method of transiently expressed plasmid transformation was performed as previously described (Yoo *et al.*, 2007). Plasmids (10 μg) and 200 μl of protoplasts were added to a 15ml tube and gently mixed. PEG solution (200 μl) was then added and incubated at room temperature for 10min. The PEG solution that contained protoplasts was diluted with 1ml of W5 solution and gently mixed. Protoplasts were centrifuged at 100 *g* (Kubota 2420) to pellet the protoplasts for 3min. The supernatant was removed, and the protoplasts were washed twice with W5 solution. The protoplasts were resuspended with 1ml of W5 solution in each well of a 6-well tissue culture plate and incubated at room temperature. After 12–16h, yellow fluorescent protein (YFP) fluorescence was detected with a confocal microscope (SP5, Leica, Microsystems, Germany). Plasmid maps are shown in Supplementary Fig. S1 at *JXB* online.

### Plasmid construction of bimolecular fluorescence complementation (BiFC) and transformation of plasmids for BiFC analysis

BiFC analyses were performed according to a modified method described previously (Yoo *et al.*, 2007; Lee *et al.*, 2012). The ORF of AtACS7 was amplified using designated primers from cDNA. The amplified ORF was inserted into the pEarleyGate201-YN vector or pEarleyGate202-YC driven by the 35S promoter and fused to YFP-N or YFP-C in-frame. Plasmids (10 μg; YFP-N and YFP-C) and 200 μl of protoplasts were added to a 15ml round-bottomed tube and gently mixed. PEG solution (110 μl) was added and incubated at room temperature for 10min. The PEG solution that contained protoplasts was diluted with 550 μl of W5 solution and gently mixed. Protoplasts were centrifuged at 100 *g* (Kubota 2420) to pellet the protoplasts for 3min. The supernatant was removed, and the protoplasts were washed twice with W5 solution. Protoplasts were resuspended with 1ml of W5 solution in each well of a 6-well tissue culture plate and incubated at room temperature. After 12–16h, YFP fluorescence was detected by a confocal microscope. Plasmid maps are shown in Supplementary Fig. S1 at *JXB* online.

### Site-directed mutagenesis

Site-directed mutagenesis was performed using the QuikChange Lightning kit (Stratagene, La Jolla, CA, USA). Two complementary oligonucleotides that contained the desired mutation, flanked by an unmodified nucleotide sequence, were designed. Mutated nucleotides were amplified by PCR. The *Dpn*I restriction enzyme (2 μl) was then directly added to each amplification reaction and incubated at 37 °C for 5min to digest the parental supercoiled double-stranded DNA. The DNA treated with 2 μl of *Dpn*I was transformed into the DH5α competent cells.

### SDS–PAGE

SDS–PAGE was performed according to a modified method described previously (Laemmli, 1970). A gel preparation system (Bio-Rad) was used to prepare a 4% stacking gel and 12% resolving gel. The samples were supplemented with sample buffer, heated at 95 °C for 5min, and subjected to SDS–PAGE at a constant voltage of 100V until the protein dye left the gel.

### Quartz crystal microbalance (QCM) sensor washing and analysis

QCM was performed according to a modified method described previously (Matsunaga and Ueda, 2010). A volume of 500 μl of 1% SDS was added to the sensor and left for 3min. Double-distilled H_2_O (ddH_2_O) was used to wash the sensor. Piranha solution (3 μl; 99% H_2_SO_4_:30% H_2_O_2_, 3:1) was added to the sensor and left for 5min. ddH_2_O was used to wash the sensor. This procedure was repeated twice. The sensor was placed into an AFFINIX QN μ (INITIUM, Japan), and the basic frequency was measured. A volume of 500 μl of PBS buffer was added to the sensor, and the frequency was left to stabilize. 6His-SUMO–ACS7 protein was added to the sensor until the sensor coating was saturated. The sensor was washed twice with PBS buffer, and 500 μl of PBS was added to the sensor. 14-3-3ω protein was added to the sensor, and the dissociation constant *K*_d_ was measured.

### Genomic DNA extraction

The leaves of 3-week-old plants were excised and placed into a 1.5ml tube with liquid nitrogen. The leaves were ground into a powder, and 750 μl of Genomic DNA extraction buffer and 50 μl of 20% SDS were added. The 1.5ml tube was heated at 65 °C for 10min. After heating, 250 μl of 5M potassium acetate was added and placed on ice for 10min. Chloroform (200 μl) was added into the 1.5ml tube and centrifuged for 10min at 13 000rpm. A total of 800 μl of the supernatant was moved to a new 1.5ml tube. Isopropanol (560 μl) was added and centrifuged at 13 000rpm for 10min. The pellet was washed with 70% ethanol and resuspended in ddH_2_O. Genomic DNA was used to determine the homozygosity of the T-DNA insertion in the *acs7-1* mutant using PCR.

### RNA extraction

RNA was isolated using REzol™ C&T reagent (Protech, Taipei, Taiwan) and converted to cDNA using a high-capacity cDNA reverse transcription kit (Applied Biosystems, Foster City, CA, USA). The leaf sample was ground into a powder with liquid nitrogen, and 1ml of REzol™ C&T and 200 μl of chloroform were added. The sample was centrifuged at 13 000rpm for 15min and moved to a new 1.5ml tube. Isopropanol (500 μl) was added and centrifuged at 13 000rpm (Sigma 1-15K, St Louis, MO, USA) for 10min. The pellet was washed with 75% ethanol and resolved by diethylpyrocarbonte (DEPC)-treated H_2_O. Total RNA (2 μg) was subjected to cDNA synthesis. The same amount of cDNA was used for PCR analysis.

### Root curvature and gravity-sustaining response ratio measurement

The root gravity response was measured as previously described (Sukumar *et al.*, 2009) (Supplementary Fig. S2 at *JXB* online). Three-day-old seedlings were transferred to either control agar (0.8%) or agar supplemented with multiple chemicals [i.e. EGTA (Sigma), LaCl_3_, LiCl, ACC, 1-naphthylphthalamic acid (NPA), and ruthenium red (RR)] at the indicated concentrations. After 12–24h of vertical growth, the plates were rotated 90 ° counterclockwise. Photographs of the plants were taken at specific time points (Time 0) after reorientation using a digital camera. The root tip curvature (in degrees) after reorientation (the angle difference of before and after reorientation) was measured every 12h by ImageJ software, and the gravity-sustaining response ratio was determined using the percentage of roots that grew toward the direction of the new gravity vector.

## Results

### AtACS7 can be phosphorylated by AtCDPK16 in vitro

**Fig. 1.**
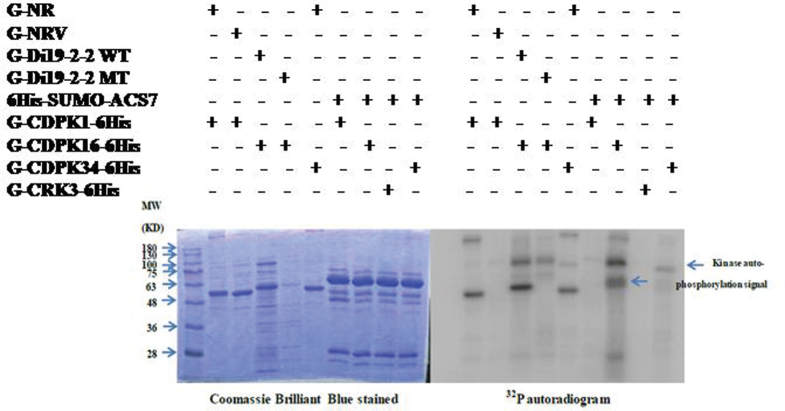
SUMO–AtACS7 was phosphorylated by AtCDPK16 *in vitro.* Recombinant protein 6His-SUMO–ACS7 was used as a substrate, and four kinds of recombinant kinases (G-AtCDPK1-6His, G-AtCDPK16-6His, G-AtCRK3-6His, and G-AtCDPK34-6His) were used to perform the kinase assay *in vitro*. The result indicates that 6His-SUMO–AtACS7 can only be phosphorylated by G-AtCDPK16-6His *in vitro.* G-NR is a fusion protein, with GST fused with a peptide of nitrate reductase (TLKRTASTPFM), and this peptide is known to be recognized by G-AtCDPK1-6His; G-NRV is a vector-only protein; G-Di19-2-2WT is also a fusion protein in which GST is fused with a peptide (DVLKSEQKEMSYREDPY); this peptide can be recognized by G-AtCDPK16-6His, and G-Di19-2-2MT is similar to G-Di19-2-2WT but with serine mutated to alanine. The molecular weights of all the fusion proteins are ~55kDa and that of SUMO–ACS7 is ~63kDa; the peptide phosphorylation signal is marked with a arrowhead, and the kinase autophosphorylation signal is ~100kDa (right panel).

A recent study found that type I and type II ACS proteins can be CDPK substrates (Sebastià *et al.*, 2004). Although the type III ACC synthase ACS7 has a shorter C-terminal domain and does not have a predicted CDPK phosphorylation site, ACS7 may still be a substrate of CDPK. AtCDPK1, AtCDPK16, AtCRK3, and AtCDPK34 were used for the *in vitro* kinase assay against the full-length ACS7 recombinant protein 6His-SUMO–AtACS7. G-NR, G-Di19-2-2 WT, and G-Di19-2-2 mutant (MT) fusion peptides were used as controls for the kinase assay as described previously (Curran *et al.*, 2011). The kinase assay results indicated that CDPK16 can phosphorylate the recombinant protein 6His-SUMO–AtACS7 *in vitro* (Fig. 1).

**Fig. 2.**
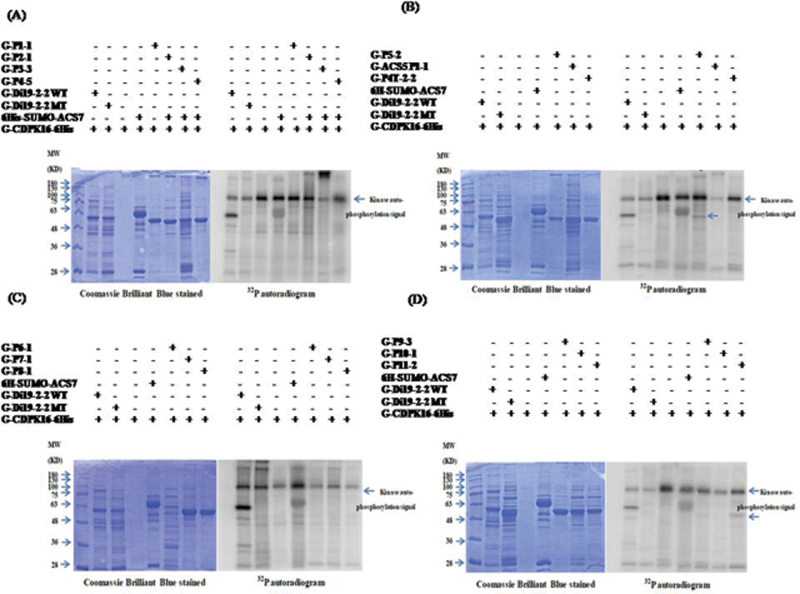
Peptide P5-2 and P11-2 were phosphorylated by CDPK16 *in vitro*. The fusion proteins (GST fused with small peptides) were designed to perform the kinase assay and determine which serine or threonine can be phosphorylated by G-CDPK16-6His. P5-2 contains the peptide V(294)GTIYSYNDNV(304); and P11-2 contains the peptide V(207)RGVLITNPS(216)NPL(219). G-Di19-2-2WT phosphorylated by G-CDPK16-6His is a positive control; G-Di19-2-2MT is a negative control; and 6His-SUMO–ACS7 is a full-length ACS phosphorylation control. The results indicate that P5-2 (in B) and P11-2 (in D) can be phosphorylated by CDPK16. The molecular weight of the fusion peptide is ~55kDa; peptide phosphorylation signals are marked with arrowheads, and the kinase autophosphorylation signal is ~100kDa (right panels).

The possible phosphorylation sites in ACS7 were further investigated. Liquid chromatography–mass spectrometry/mass spectrometry (LC-MS/MS) was used to identify the peptides in the ACS7 recombinant protein phosphorylated by CDPK. According to the LC-MS/MS results and conserved Ser/Thr residues of ACS in different plant species, 13 fusion peptides that contained possible phosphorylation sites were designed as candidates for the *in vitro* kinase assays (Supplementary Table S1 at *JXB* online). After the kinase assay, it was found that the fusion peptides P5-2, which contains the peptide V(294)GT(296)IYS(299)YNDNV(304), and P11-2, which contains the peptide V(207)RGVLIT(213)NPS(216)NPL(219), were labelled in the ^32^P autoradiogram (Fig. 2). This result indicates that P5-2 and P11-2 can be phosphorylated by CDPK16 *in vitro*.

**Fig. 3.**
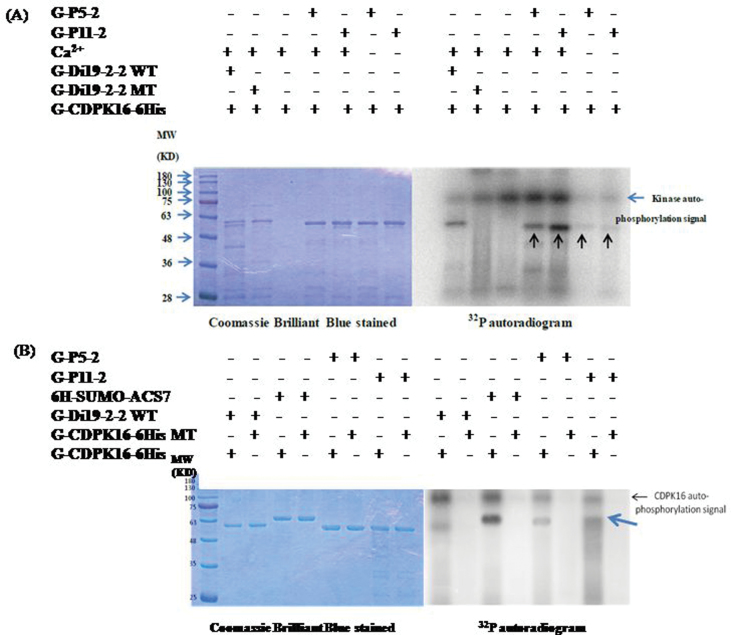
The phosphorylation of P5-2 and P11-2 is calcium dependent. (A) In order to confirm that this phosphorylation reaction is calcium dependent, buffer with or without calcium ions was tested. The result indicated that the substrate kinase phosphorylation reaction is calcium dependent (lanes 4–7). The amino acid sequences in P5-2 and P11-2 are VGTIYSYNDNV and VRGVLITNPSNPL. Peptide phosphorylation signals are marked with arrowheads. (B) An *in vitro* kinase assay was carried out using mutated G-CDPK16-6His (G-CDPK16-6His MT) having double mutations on both Ser274 and Ser541. Both serines were mutated to alanine. The AtACS7 fusion protein and peptide phosphorylation signals are marked with arrowheads.

A previous study showed that CDPK phosphorylates substrates in a calcium-dependent manner (Hetherington and Trewavas, 1984). Because the fusion peptides P5-2 (VGTIYSYNDNV) and P11-2 (VRGVLITNPSNPL) were shown to be phosphorylated by CDPK16, experiments were carried out to determine whether this phosphorylation is calcium dependent. Figure 3A shows that when calcium ions were added in the kinase reaction buffer, P5-2 and P11-2 phosphorylation signals were detected. When calcium ions were depleted in the reaction buffer, phosphorylation was abolished. This confirms that P5-2 and P11-2 phosphorylation is calcium dependent.

**Fig. 4.**
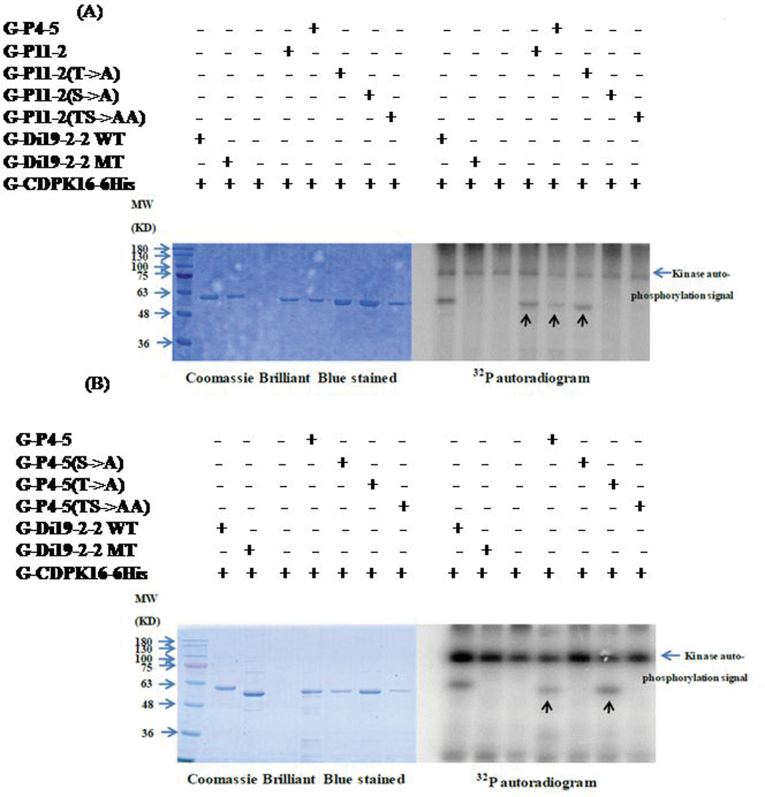
Phosphorylation of Ser216 was confirmed by site-directed mutagenesis. In order to identify the phosphorylation residue in P11-2, site-directed mutagenesis was performed. P4-5 is a peptide which has a 10 amino acid sequence overlapping with P11-2. P11-2 S→A is a serine to alanine mutation peptide in P11-2; P11-2 T→A is a threonine to alanine mutation peptide; P11-2 TS→AA is a double mutation peptide of serine and threonine to alanine. The results indicated that only serine (Ser216) but not threonine (Thr213) in P11-2 can be phosphorylated by G-CDPK16-6His *in vitro*. Peptide phosphorylation signals are marked with arrowheads.

To confirm that the *in vitro* phosphorylation of AtACS7 fusion peptides was due to AtCDPK16 activity, a control experiment was carried out. Two autophosphorylation sites (Ser274 and Ser541) of the AtCDPK16 recombinant fusion protein (Hegeman *et al.*, 2006) were mutated by site-directed mutagenesis. The mutated AtCDPK16 recombinant fusion protein (G-CDPK16-6His MT) was used in the *in vitro* kinase assay against AtACS7 fusion peptides. The results showed that the autophosphorylation of the mutated recombinant protein (G-CDPK16-6His MT) was greatly reduced (Fig. 3B; Supplementary Fig. S3 at *JXB* online). In addition, the phosphorylation of the AtACS7 fusion peptides was greatly reduced (Fig. 3B; Supplementary Fig. S3). This indicated that the phosphorylation of AtACS7 fusion peptides *in vitro* resulted from AtCDPK16 but not from other bacterial kinases. The P5-2 and P11-2 peptides each contain one serine and one threonine residue. Furhter experiments were carried out to determine which amino acid is the target for CDPK16 or whether both can be phosphorylated by CDPK16. To address this issue, a point mutation was created in the two peptides using site-directed mutation of either serine or threonine to alanine (Supplementary Table S1 at *JXB* online). For P11-2, the results indicated that only Ser216 was recognized by CDPK16 (Fig. 4). To confirm the phosphorylation sites, the fusion peptide P4-5, which has 10 amino acids that overlap with P11-2, was used for the kinase assay. A point mutation of either serine or threonine to alanine was designed to verify the phosphorylation site in P11-2. The results showed that only Ser216 was recognized by CDPK16 (Fig. 4B). This result was consistent with the results presented in Fig. 4A. The serine residue (Ser216) in both P11-2 and P4-5 was shown to be an AtCDPK16 phosphorylation site.

**Fig. 5.**
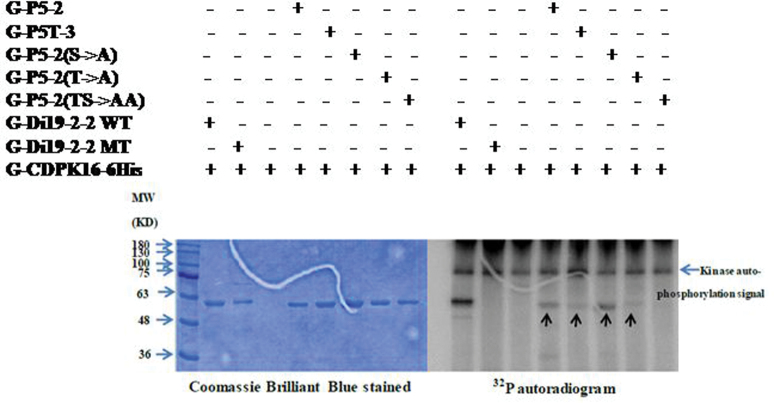
Site-directed mutagenesis of the hosphorylation site on peptide P5-2. In order to identify the phosphorylation residue in P5-2, site-directed mutagenesis was performed. P5T-3 is a peptide which has a nine amino acid sequence overlapping with P5-2. P5-2 S→A is a peptide with a mutation of serine (Ser299) to alanine in P5-2; P5-2 T→A is a threonine (Thr296) to alanine mutation peptide; P5-2 TS→AA is a double mutation peptide of serine and threonine to alanine. The results indicated that both serine and threonine in P5-2 can be phosphorylated by G-CDPK16-6His *in vitro*. Peptide phosphorylation signals are marked with arrowheads.

For P5-2, a fusion peptide with a single point mutation of either threonine or serine to alanine and double mutations of both serine and threonine to alanine were used to perform the kinase assay. The results showed that both Thr296 and Ser299 were recognized by CDPK16 (Fig. 5). Specifically, the phosphorylation of Ser299 was also supported by the MS/MS data (Supplementary Fig. S4 at *JXB* online). In conclusion, based on the *in vitro* assay, three AtCDPK16 phosphorylation sites, Ser216, Thr296, and Ser299, were identified. These results suggest that AtACS7 may be involved in calcium signalling.

### The subcellular localization of AtACS7 is in the cytosol

**Fig. 6.**
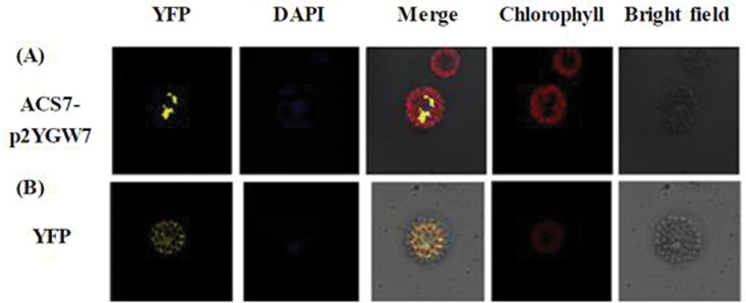
Subcellular localization of AtACS7 in *Arabidopsis* protoplasts in a transient expression assay. Wild-type *Arabidopsis* protoplasts were transfected with (A) 35S:YFP:AtACS7 and (B) 35S:YFP (control) constructs. YFP signals were observed by confocal microscopy. The blue signal showed a nucleus stained with 4′,6-diamidino-2-phenylindole (DAPI); the red signal showed chlorophyll with autofluorescence; and merge shows YFP, DAPI, chlorophyll, and bright field signals.

ACS proteins have been found to be cytosolic proteins (Yip *et al.*, 1991). To confirm further whether AtACS7 is actually localized in the cytosol, transient expression of ACS7 protein in protoplasts was detected. The 35S::YFP:ACS7 plasmid was transferred to *Arabidopsis* protoplasts, and confocal microscopy was used to observe the localization of the recombinant YFP fused with ACS7. As expected, ACS7 protein was localized in the cytosol based on the transient assay (Fig. 6).

### Protein–protein interaction between ACS7 and 14-3-3ω was confirmed using BiFC and QCM

14-3-3 proteins have been regarded as scaffold proteins that can bind to phosphorylated proteins (Sehnke *et al.*, 2002). A previous study showed that ACS7 can be a client of 14-3-3ω in *Arabidopsis* (Chang *et al.*, 2009; Yoon and Kieber, 2013). Whether 14-3-3ω physically interacts with ACS7 and modulates its functions was investigated. Previous studies showed that ACS7 can form a homodimer to perform its functions (Tsuchisaka and Theologis, 2004), and fusion proteins ACS7-YN and ACS7-YC were used as a positive control (Supplementary Fig. S5B at *JXB* online). ACS7 formed homodimers as expected (Tsuchisaka and Theologis, 2004). Additionally, NR peptide, which contains a 14-3-3 interaction site, interacted with 14-3-3ω as expected. Based on the transient expression results, ACS7 physically interacted with 14-3-3ω in the cytosol (Supplementary Fig. S5E, F). Both ACS7-YN and ACS7-YC interacted with 14-3-3ω-YN.

**Fig. 7.**
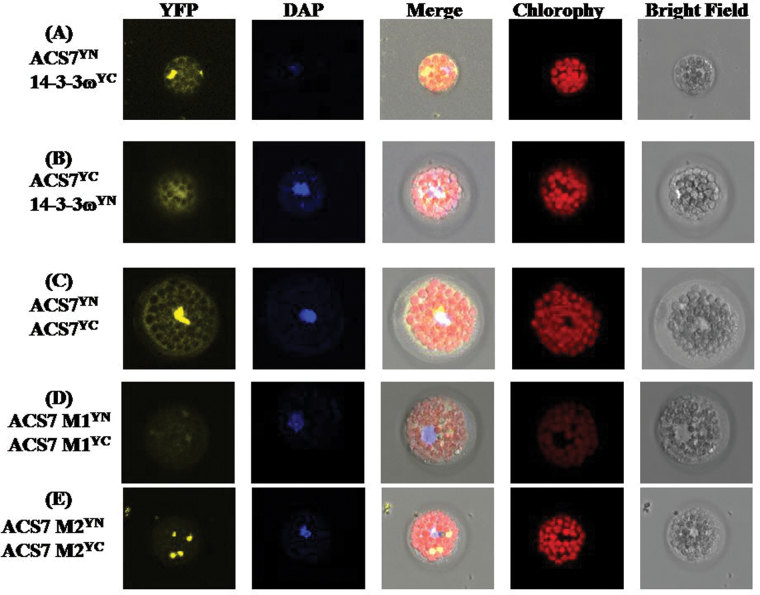
Protein–protein interactions with ACS7. (A) Co-expression of NR-nEYFP and 14-3-3-cEYFP (positive control). (B) Co-expression of NR-cEYFP and 14-3-3-nEYFP (positive control). (C) Co-expression of ACS7-nEYFP and 14-3-3cEYFP. (D) Co-expression of ACS7-M1. (E) Co-expression of ACS7-M2. (D) and (E) are ACS7 mutant proteins for testing ACS7 homodimer formation. M1: Thr296 and Ser299 of AtACS7 were both mutated to alanine. M2: Ser216 of AtACS7 was point mutated to alanine. YFP signals were observed by confocal microscopy. The blue signal shows a nucleus stained with 4′,6-diamidino-2-phenylindole (DAPI); the red signal showed chlorophyll with autofluorescence; and merge shows YFP, DAPI, chlorophyll, and bright field signals.

To investigate the functional relevance of the identified phosphorylation sites, ACS7-YN and ACS7-YC, each with Ser216 point-mutated to alanine, were tested for interactions using BiFC. ACS7-YN and ACS7-YC, each with Thr296 and Ser299 double-mutated to alanine, were also tested. Both BiFC results showed that the mutation of these phosphorylation sites resulted in an altered subcellular localization pattern. The dimer formation signal was weaker and not evenly distributed in the entire cytoplasm as it was in the WT (Fig. 7C–E), suggesting that the phosphorylation of AtACS7 may have important functions.

To confirm further the protein–protein interaction between ACS7 and 14-3-3ω, QCM analysis was performed. The recombinant protein G-14-3-3ω was first coated on the sensor, and then 6His-SUMO–ACS7 protein was injected into the PBS buffer. If the recombinant protein 6His-SUMO–ACS7 can physically interact with G-14-3-3ω, then the sensor frequency would change. The results showed that 6His-SUMO–ACS7 interacted with G-14-3-3ω *in vitro* (Supplementary Fig. S6A at *JXB* online). The dissociation constant (*K*_d_) was 9.29±1.99×10^–9^ M (Supplementary Fig. S6C). A previous study showed that 14-3-3 is a scaffold protein that can interact with its clients that have already been phosphorylated (Wu *et al.*, 1997). To determine whether the interaction between ACS7 and 14-3-3ω is phosphorylation dependent and whether the interaction is caused by ACS7 phosphorylation catalysed by CDPK16, an interaction assay between phosphorylated 6His-SUMO–ACS7 and G-14-3-3ω was performed. 6His-SUMO–ACS7 was phosphorylated by CDPK16 *in vitro* in advance. The results showed that phosphorylated 6His-SUMO–ACS7 can physically interact with G-14-3-3ω *in vitro* (Supplementary Fig. S6B), and the *K*_d_ was 5.05±1.96×10^–9^ M (Supplementary Fig. S6D). The QCM results of tag-only controls did not show any interaction, as expected (Supplementary Fig. S6E, F). Altogether, the results are consistent with the previous discovery that ACS7 forms a protein complex with 14-3-3ω protein (Chang *et al.*, 2009; Yoon and Kieber, 2013). The interaction between ACS7 and 14-3-3ω protein appeared to be both phosphorylation dependent and phosphorylation independent.

### Identification of acs7-1 loss-of-function mutant line

To study the functions of AtACS7 *in planta*, an *acs7-1* mutant line was ordered from the Arabidopsis Biological Research Center (http://abrc.osu.edu/). In this mutant line, T-DNA was inserted into the third exon of the *AtACS7* gene (Supplementary Fig. S7A at *JXB* online). The background of the mutant line was Wassilewskija (WS). Genomic PCR was used to confirm the T-DNA insertion site. The T-DNA primer GBK5-F paired with the gene primer acs7-1-R, and the gene primer acs7-1-F paired with acs7-1-R were used to perform PCR. It was found that only the T-DNA primer GBK5-F with acs7-1-R flanked the DNA fragment, and acs7-1-F with acs7-1-R did not (Supplementary Fig. S7B). The results showed that the *acs7-1* mutant was homozygous.

### The acs7-1 mutant is less sensitive to inhibition by a calcium chelator and the channel blocker LiCl in the gravity response

**Fig. 8.**
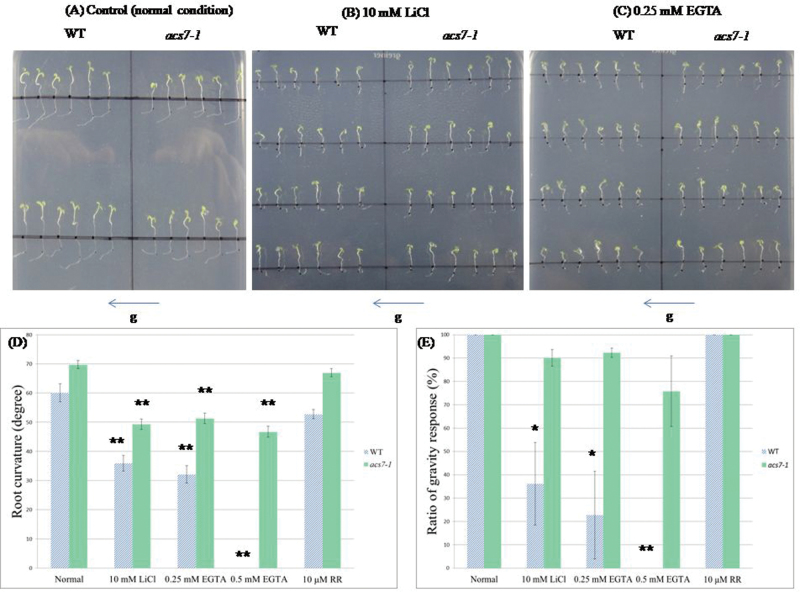
Multiple calcium chelator and channel blockers affected the gravity response in *acs7-1.* The WT (WS) and the *acs7-1* mutant were grown in half-strength Murashige and Skoog (1/2MS) medium for 3 d and transferred to 1/2MS medium which contained (A) control (normal conditions), (B) 10mM LiCl, (C) 0.25mM EGTA and other calcium channel blockers, and grown for 1 d. The gravity vector is downwards as the arrowhead indicates. The root curvature (degree) and ratio of gravity response (%) were measured in both the WT and the *acs7-1* mutant after 24h (D and E). The average and SE are presented (*n*=60) for multiple calcium chelators or channel blockers in three independent experiments in (C), and the ratio of the gravity response is presented (*n*=60) for multiple calcium chelators or channel blockers in (D). RR, ruthenium red. Significant differences between normal conditions and treatment groups are indicated by * at *P* < 0.05 and by ** at *P* < 0.01 by Student’s *t*-test.

A previous study indicated that *acs7-1* has multiple phenotypes (i.e. lower ethylene emission, larger cotyledons and true leaves, and longer primary root length; Dong *et al.*, 2011). Additionally, the *acs7-1* mutant exhibited hypersensitivity to abscisic acid (ABA) during seed germination (Dong *et al.*, 2011). However, no studies of which the authors are aware have discussed the aspect of calcium in the *acs7-1* mutant. The phenotype of the *acs7-1* mutant line has been widely described (Tsuchisaka *et al.*, 2009; Dong *et al.*, 2011). The *acs7-1* mutant exhibits early flowering, slightly reduced ethylene production, larger cotyledons, and enhanced salt tolerance. Because little is known about the relationship between ethylene biosynthesis and calcium (Raz and Fluhr, 1992; Philosoph-Hadas *et al.*, 1996), several calcium channel blockers (i.e. LiCl, LaCl_3_, and RR), a calcium chelator (i.e. EGTA), and the CaM antagonist chlorpromazine (CPZ) were used to investigate the relationships between them. AtACS7 can be phosphorylated by CDPK *in vitro*, and it is hypothesized that AtACS7 may be involved in calcium signalling. Previous studies showed that calcium ions are important for the gravity response. When the WT seedlings are treated with calcium channel blockers or a calcium chelator, the plants may lose their gravity response (Hasenstein and Evans, 1988; Friedman *et al.*, 1998; Toyota *et al.*, 2008). To investigate the relationship between *acs7-1* and calcium, the calcium channel blockers LiCl, LaCl_3_, and RR, the calcium chelators EGTA, and the CaM antagonist CPZ were used to determine whether calcium ions influence *acs7-1*. The results indicated that LaCl_3_ and CPZ did not cause significant differences between the WT and the *acs7-1* mutant (Supplementary Figs S8, S9 at *JXB* online). Surprisingly, EGTA treatment caused a loss of the gravity response in the WT but not in the *acs7-1* mutant. Figure 8C–E shows that *acs7-1* can retain gravitropism, but the WT almost lost gravitropism. Moreover, 10mM LiCl treatment had the same effect as EGTA treatment (Fig. 8B, D, E). Collectively, the results indicate that ACS7 is involved in root gravitropism in a calcium-dependent manner in *Arabidopsis*.

### The acs7-1 mutant is less sensitive to ethylene in the triple response

**Fig. 9.**
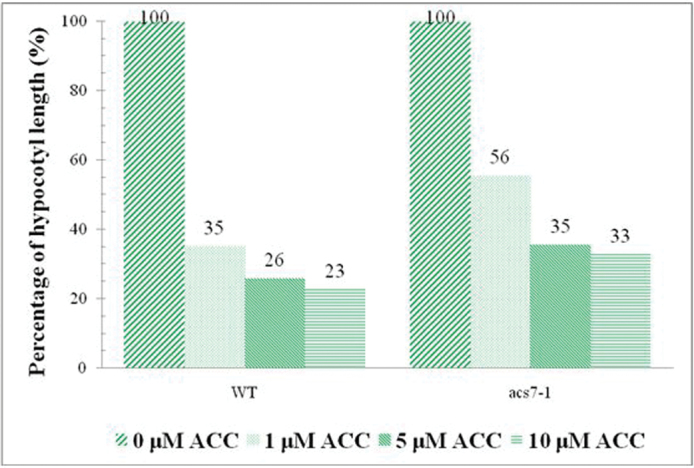
Triple response in theWT and *acs7-1* mutant. The experiment was performed at 22°C for 5 d in the dark. Hypocotyl length and the percentage of hypocotyl length relative to normal conditions were measured by ImageJ. The average and SE are presented (*n*=60) for ACC in three independent experiments. A higher concentration of ACC in the dark shows reduced hypocotyl length more in the WT than in the *acs7-1* mutant.

To determine whether the *acs7-1* mutant displays ethylene-related phenotypes that are different from the WT in etiolated seedlings, the hypocotyl length was measured in the dark for 5 d and analysed by ImageJ software. The triple-response results showed that the *acs7-1* mutant is less sensitive to ethylene compared with the WT (Fig. 9).

### Gravity response under optimal ACC concentration

**Fig. 10.**
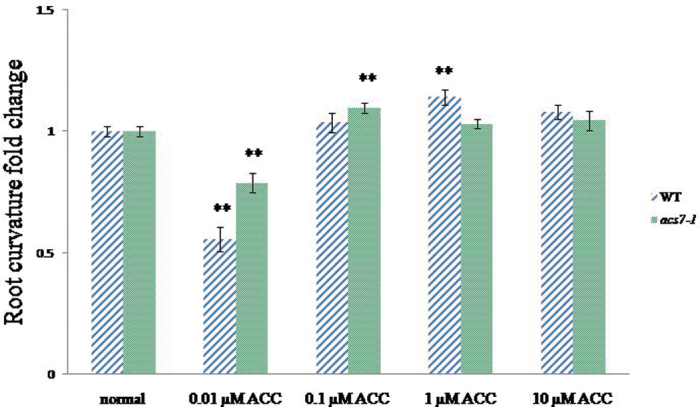
ACC gradient for gravity response. The WT (WS) and *acs7-1* mutant were grown in half-strength Murashige and Skoog medium for 3 d and transferred to medium which contained 0.01, 0.1, 1, or 10 μM ACC, and the root curvature of the root tip was measured by ImageJ after a 12h change of the gravity vector (turned counterclockwise by 90 °). Significant differences between normal conditions and treatment groups are indicated by ** at *P* < 0.01 by Student’s *t*-test.

Ethylene has been suggested to regulate the gravity response. Some studies indicate that it plays a negative role in gravitropism (Buer *et al.*, 2006), but other studies suggest a positive role in gravibending (Philosoph-Hadas *et al.*, 1996). To investigate the effects of ethylene on gravitropism, gradient concentrations of ACC were introduced in the medium. Both the WT and the *acs7-1* mutant responded to certain concentrations of ethylene that positively regulated the gravity response (WT at 1 μM ACC; *acs7-1* at 0.1 μM ACC). A specific concentration of ethylene with 0.01 μM ACC negatively regulated the gravity response in both the WT and the *acs7-1* mutant (Fig. 10).

### The acs7-1 mutant is less sensitive to NPA-mediated inhibition of the gravity response

**Fig. 11.**
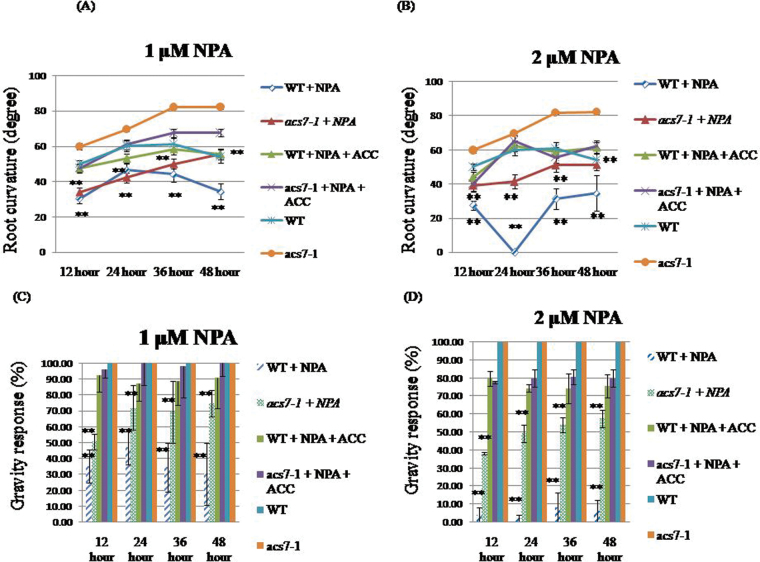
Root curvature and the ratio of the gravity response were affected by NPA in root tips. The WT (WS-4) and *acs7-1* mutant were grown in half-strength Murashige and Skoog (1/2MS) medium for 3 d and transferred to medium which contained 1 μM or 2 μM NPA (A–D); the percentage gravity response was measured every 12h for 2 d. The percentages compared with normal conditions are presented (*n*=36) in three independent experiments. For ACC treatment, the WT (WS-4) and *acs7-1* mutant were grown in 1/2MS medium for 3 d and transferred to medium which contained 1 μM or 2 μM NPA combined with 0.1 μM ACC (A and B); the root tip curvature was measured every 12h for 2 d by ImageJ software. The average is presented (*n*=36) in three independent experiments. The percentage gravity response was measured every 12h for 2 d. The percentage compared with normal conditions is presented (*n*=36) in three independent experiments (C and D). Significant differences between normal conditions and NPA treatment groups are indicated by ** at *P* < 0.01 by Student’s *t*-test.

Some auxin polar transport inhibitors, including NPA and 2,3,5-triiodobenzoic acid (TIBA), were found to inhibit auxin polar transport. For example, TIBA can compete with auxins for translocation across the plasma membrane (Depta and Rubery, 1984), and NPA was found to disturb the polarity of cell division (Thomson *et al.*, 1973). Therefore, NPA has long been used as an inhibitor of auxin polar transport (Thomson *et al.*, 1973). The gravity response is greatly reduced by NPA treatment. To investigate the effects of NPA on the gravity response in the WT and the *acs7-1* mutant, different concentrations (12 μM and 2 μM) of NPA were tested. The WT showed a smaller root curvature and lower gravity response than the *acs7-1* mutant (Fig. 11A–D) in response to NPA treatment. This indicates that the *acs7-1* mutant was less sensitive to the inhibitory effect of NPA. Exogenous ACC (0.1 μM) was added to the medium with NPA, which greatly reduced the effect of NPA and recovered root gravitropism (Fig. 11A–D). These results suggest that AtACS7 is involved in the root gravity response through an unknown cross-talk relationship between auxin and ethylene.

## Discussion

Figure 1 shows the phosphorylation of AtACS7 catalysed by AtCDPK16 *in vitro*. However, this result is different from the previous expectation that type III ACS proteins have no predicted CDPK phosphorylation sites and may not be a substrate of CDPKs. To date, only one *Arabidopsis* AtCDPK16 substrate, AtDi19-2, has been documented (Curran *et al.*, 2011). The present study showed that AtCDPK16 can phosphorylate AtACS7 *in vitro*, but whether AtACS7 is an AtCDPK16 substrate requires further studies. Several consensus CDPK phosphorylation motifs are known (Harper and Harmon, 2005). The first consensus phosphorylation motif is ϕ_-5_-X_-4_-Basic_-3_-X_-2_-X_-1_-*S*, in which the underlined S is phosphorylated, X is any residue, and ϕ is a hydrophobic residue (Huang and Huber, 2001). The second consensus phosphorylation motif is Basic_-9_-Basic_-8_-X_-7_-Basic_-6_-ϕ_-5_-X_-4_-X_-3_-X_-2_-X_-1_-*S*-X_+1_-Basic_+2._ The third consensus phosphorylation motif is ϕ_-3_-R_-2_-ϕ_-1_-*S*-ϕ_+1_-x-K_+3_-R_+4_, which was defined as an ACS motif in a previous study (Sebastià *et al.*, 2004). The identified phosphorylation site of AtACS7, Thr296, perfectly matches the first consensus phosphorylation motif. The phosphorylation motif S-X-R was recently identified from the AtCDPK16 substrate AtDi19-2 (Curran *et al.*, 2011). In the present study, Ser216 and Ser299 were found to be phosphorylated by CDPK16 in ACS7 *in vitro* (Figs 2, 4, 5) but did not conform to any of these consensus phosphorylation motifs. Ser216 and Ser299 appear to be newly identified CDPK phosphorylation sites with an unknown CDPK phosphorylation motif. Whether Ser216, Thr296, and Ser299 are indeed phosphorylated by CDPK *in vivo* needs further confirmation.

The *Arabidopsis* genome has eight genes that encode active ACS proteins and an additional gene that encodes a catalytically inactive enzyme, ACS1 (Liang *et al.*, 1992). Previous studies showed that ACS1 was inactive because it lacked the highly conserved tripeptide Thr-Asn-Pro (TNP) between Ile204 and Ser205 in the ACS conserved region that was responsible for binding the cofactor PLP (Liang *et al.*, 1995; Yamagami *et al.*, 2003). The introduction of TNP into ACS1 restores ACS activity, whereas its removal from enzymatically active ACS2 results in a loss of activity (Liang *et al.*, 1995). In the sequence alignment of plant ACS proteins (data not shown), it was found that ACS7 has a tripeptide, Thr-Asn-Pro (TNP), between Ile212 and Ser216. Ser216 was phosphorylated by CDPK16 in the *in vitro* kinase assay (Figs 2, 4), and the phosphorylation of Ser216 in ACS7 can probably influence the interaction between ACS7 and its cofactor PLP and finally change the enzyme activity. To investigate whether phosphorylation can influence enzyme activity, an *in vitro* enzyme activity assay was performed according to previous studies (Li *et al.*, 1996; Chae *et al.*, 2003). The results indicated that non-phosphorylated ACS7 had a *K*_m_ value of 27.43 μM, whereas phosphorylated ACS7 had a lower *K*_m_ value (13.65 μM; data not shown). These results suggest that phosphorylated ACS7 has a higher catalytic ability, and the phosphorylation of ACS7 catalysed by CDPK16 may enhance ACS7 activity. However, such a possibility requires further studies.

ACS gene expression in light-grown mature *Arabidopsis* plants was previously studied using reverse transcription–PCR (RT–PCR) (Yamagami *et al.*, 2003), in which the expression of all *Arabidopsis ACS* genes was surveyed in plant roots, leaves, stems, flowers, and siliques. These researchers found that *ACS7* was expressed only in roots. It was confirmed here that ACS7 is a cytosolic protein (Fig. 6), suggesting that ACS7 protein is mainly localized in the root cytosol. Another study used a green fluorescent protein (GFP) fused to nine kinds of AtCDPKs (Dammann *et al.*, 2003). The authors found that AtCDPK16 was localized to the root plasma membrane in a thin layer at the periphery of the cell. Further studies are needed to confirm whether ACS7 can be phosphorylated by CDPK16 in plant roots *in vivo*.

Lithium chloride is known to suppress root curvature in *Pisum sativum* seedlings (Belyavskaya, 2001). In *Arabidopsis*, the root cap comprises four tiers of columella cells that originate from initial columella cells. Columella cells have a specific polarity, in which the nucleus and ER are localized to the proximal side of the root meristem and periphery of the cell, respectively. In addition to the peripheral arrangement of the ER, its function as an intracellular calcium reservoir has been the preferred hypothesis for a possible gravity-sensing mechanism in columella cells, in which amyloplasts that come into contact with the peripheral ER can trigger the release of calcium stored in the ER (Perbal and Driss-Ecole, 2003). Many signalling molecules and secondary messengers, such as calcium (Plieth and Trewavas, 2002) and InsP_3_ (Perera *et al.*, 2001), and pH (Johannes *et al.*, 2001) have been implicated in linking gravity sensing to the initiation of a differential growth response. Very rapid gravity-specific changes in transcript abundance have been documented in gravistimulated root tips of *Arabidopsis* (Kimbrough *et al.*, 2004) and whole seedlings (Moseyko *et al.*, 2002). InsP_3_ is well known to induce calcium release from intracellular calcium reservoirs. Changes in InsP_3_ levels have been reported after the gravistimulation of pulvini in maize (Perera *et al.*, 1999). Lithium chloride has been reported to be a phosphatidylinositol cycle inhibitor (Belyavskaya, 2001; Dieck *et al.*, 2012). It can suppress InsP_3_ cycling and signalling by inhibiting inositol-1-phosphatase (Belyavskaya, 2001), leading to a reduction in the amount of InsP_3_. When plants are treated with LiCl, some plant organs, such as tonoplasts and the ER, that are reported to have IP_3_-sensitive calcium channels may be blocked, leading to a reduction of calcium ion efflux from these organs. When the gravity vector changes, InsP_3_ may bind to the ER or tonoplast InsP_3_-sensitive calcium channels, which ultimately leads to calcium ion efflux from these organs. These calcium ions are captured by some calcium sensors, such as CDPK and CaM, and pass the signals to downstream effectors, ultimately leading to the plant gravity response.

Several mutant lines have been shown to be related to calcium signalling in root gravitropism in plants. A mutation of the calmodulin *agr-3* gene exerted a reduced root gravitropism phenotype in *Arabidopsis* (Sinclair *et al.*, 1996). Spalding’s group found that a mutation of the *Glutamate Receptor-Like 3.3* gene (i.e. a mammalian calcium channel orthologue) showed a root gravitropism phenotype (Miller *et al.*, 2010). Moreover, an *Arabidopsis* calcium-dependent binding protein *AtCLB* gene overexpression line exhibited reduced root gravitropism, but a T-DNA insertion mutant exhibited greater root gravitropism than the WT (de Silva *et al.*, 2011). However, little is known about whether the *ACS* gene is involved in calcium signalling. Additionally, these mutant lines expressed a root gravitropism phenotype without EGTA treatment. In contrast, the present study observed root gravitropism with EGTA treatment. Therefore, the root gravitropism phenotype observed here appeared to be distinct from others.

Auxin polar transport is involved in many developmental processes, such as vascular differentiation and tropic growth (Luschnig *et al.*, 1998; Mattson *et al.*, 1999; Rashotte *et al.*, 2000). The auxin polar transport inhibitor NPA was used to investigate the role of the *ACS7* gene in the gravity response using a T-DNA knockout *acs7-1* mutant line and it was found that *acs7-1* is more resistant to NPA-mediated inhibition (Fig. 11). The data indicate that the inhibition of the gravity response by NPA was greatly attenuated by the addition of 0.1 μM exogenous ACC (Fig. 11). Collectively, these findings suggest cross-talk between auxin and ethylene in root gravitropism regulated by *AtACS7*. Cross-talk between the phytohormone auxin and ethylene in root gravitropism has been reported previously. In 1990, Estelle’s group found that an *aux1* mutant exhibited a defect in root gravitropism and resistance to both auxin and ethylene in *Arabidopsis* (Pickett *et al.*, 1990). In 2003, an *alh1* mutation revealed cross-talk between ethylene and auxin in *Arabidopsis* (Vandenbussche *et al.*, 2003). However, how auxin engages in cross-talk with ethylene to regulate root gravitropism through *AtACS7* in *Arabidopsis* requires further investigation.

The rice type II ACC synthase OsACS1 was found to interact with rice 14-3-3 proteins in a yeast two-hybrid assay (Yao *et al.*, 2007). These authors found that the C-terminal domain of OsACS1 (RSVpSCP) was predicted to be phosphorylated by CDPK through a Mode-I 14-3-3 recognition motif. They suggested that OsACS1 phosphorylation catalysed by OsCDPK may interact with rice 14-3-3 proteins, which can prevent the substrate ubiquitin adaptor protein ETO1 from binding to produce 26S proteasome degradation. Interactions with 14-3-3 proteins on the CDPK phosphorylation site may prevent ETO1 protein binding, and this may be a reason why the phosphorylation of ACS proteins catalysed by CDPK enhances ACS protein stability. In fact, the phosphorylation status of AtACS6 affected the stability of AtACS6 (Skottke *et al.*, 2011). Moreover, the stability of AtACS5 was also found to be regulated by phosphorylation and 14-3-3 binding (Yoon and Kieber, 2013). A recent study found that ACS7 is turned over in a 26S proteasome-dependent manner and that ACS7 degradation requires the E3 ligase XBAT32 (Lyzenga *et al.*, 2012). Whether phosphorylation and 14-3-3 binding are involved in the regulation of AtACS7 degradation requires further investigation.

Apart from ACS, ACO is another important enzyme in the ethylene biosynthesis pathway that catalyses the oxidation of ACC to ethylene. ACO is encoded by a small multigene family that is usually composed of three to four members. In the *Arabidopsis* genome, six genes that encode ACOs were identified *in silico* by Babula *et al.* (2006). Recently, ACO2 and ACO4 proteins were found, which may interact with 14-3-3 proteins in a yeast two-hybrid system (Jaspert *et al.*, 2011). Together with the present results, this indicates that 14-3-3 proteins may regulate the ethylene biosynthesis pathway by modulating ACS and ACO proteins. A previous study showed that an ACO promoter can respond to IAA, bending stress, and gravity (Yuan and Dean, 2010). Together, ACS proteins and ACO may synergistically participate in the gravity response by regulating ethylene concentrations in plant cells.

## Supplementary data

Supplementary data are available at *JXB* online.

Figure S1. Plasmid maps used in the present study.

Figure S2. Gravity response measurement method.

Figure S3. Bar graph of the phosphorylation signal in the *in vitro* kinase assay using mutated AtCDPK16.

Figure S4. MS/MS fragmentation pattern of a phosphorylated peptide (VGTIYSYNDNVVR) of ACS7 recombinant protein.

Figure S5. Protein–protein interaction between ACS7 and 14-3-3ω using BiFC analysis.

Figure S6. Quartz crystal microbalance (QCM) and kinetic analysis of protein–protein interaction between ACS7 and 14-3-3ω recombinant proteins.

Figure S7. Isolation of the T-DNA insertional mutant *acs7-1.*

Figure S8. Gravity response of the WT and *acs7-1* mutant affected by 0.5mM LaCl_3_.

Figure S9. The effect of 50 μM CPZ on the WT and *acs7-1* mutant.

Table S1. Information on fusion peptides.

Supplementary Data

## References

[CIT0001] Adams DO Yang SF 1979 Ethylene biosynthesis: identification of 1-aminocyclopropane-1-carboxylic acid as an intermediate in the conversion of methionine to ethylene. Proceedings of the National Academy of Sciences, USA 76, 170–174 10.1073/pnas.76.1.170 PMC382898 16592605

[CIT0002] Babula D Misztal LH Jakubowicz M Kaczmarek M Nowak W Sadowski J 2006 Genes involved in biosynthesis and signalisation of ethylene in *Brassica oleracea* and *Arabidopsis thaliana*: identification and genome comparative mapping of specific gene homologues. Theoretical and Applied Genetics 112, 410–420 16311726 10.1007/s00122-005-0136-7

[CIT0003] Batistic O Kudla J 2012 Analysis of calcium signaling pathways in plants. Biochimica et Biophysica Acta 1820, 1283–1293 22061997 10.1016/j.bbagen.2011.10.012

[CIT0004] Belyavskaya NA 2001 Lithium-induced changes in gravicurvature, statocyte ultrastructure and calcium balance of pea roots. Advances in Space Research 27, 961–966 11596640 10.1016/s0273-1177(01)00173-9

[CIT0005] Benková E Michniewicz M Sauer M Teichmann T Seifertová D Jürgens G Friml J 2003 Local, efflux-dependent auxin gradients as a common module for plant organ formation. Cell 115, 591–602 14651850 10.1016/s0092-8674(03)00924-3

[CIT0006] Blakeslee JJ Bandyopadhyay A Peer WA Makam SN Murphy AS 2004 Relocalization of the PIN1 auxin efflux facilitator plays a role in phototropic responses. Plant Physiology 134, 28–31 14730061 10.1104/pp.103.031690 PMC1540349

[CIT0007] Blilou I Xu J Wildwater M Willemsen V Paponov I Friml J Heidstra R Aida M Palme K Scheres B 2005 The PIN auxin efflux facilitator network controls growth and patterning in Arabidopsis roots. Nature 433, 39–44 15635403 10.1038/nature03184

[CIT0008] Bradford MM 1976 A rapid and sensitive method for the quantitation of microgram quantities of protein utilizing the principle of protein–dye binding. Analytical Biochemistry 72, 248–254 942051 10.1016/0003-2697(76)90527-3

[CIT0009] Buer CS Sukumar P Muday GK 2006 Ethylene modulates flavonoid accumulation and gravitropic responses in roots of Arabidopsis. Plant Physiology 140, 1384–1396 16489132 10.1104/pp.105.075671 PMC1435817

[CIT0010] Chae HS Faure F Kieber JJ 2003 The *eto1*, *eto2*, and *eto3* mutations and cytokinin treatment increase ethylene biosynthesis in Arabidopsis by increasing the stability of ACS protein. The Plant Cell 15, 545–559 12566591 10.1105/tpc.006882 PMC141220

[CIT0011] Chae HS Kieber JJ 2005 Eto Brute? Role of ACS turnover in regulating ethylene biosynthesis. Trends in Plant Science 10, 291–296 15949763 10.1016/j.tplants.2005.04.006

[CIT0012] Chang IF Curran A Woolsey R Quilici D Cushman JC Mittler R Harmon A Harper JF 2009 Proteomic profiling of tandem affinity purified 14-3-3 protein complexes in *Arabidopsis thaliana* . Proteomics 9, 2967–2985 19452453 10.1002/pmic.200800445 PMC4077669

[CIT0013] Chen R Hilson P Sedbrook J Rosen E Caspar T Masson PH 1998 The *Arabidopsis thaliana* AGRAVITROPIC 1 gene encodes a component of the polar-auxin-transport efflux carrier. Proceedings of the National Academy of Sciences, USA 95, 15112–15117 10.1073/pnas.95.25.15112 PMC24584 9844024

[CIT0014] Crocker W Knight LL 1908 Effect of illuminating gas and ethylene upon flowering carnations. Botanical Gazette 46, 259–276

[CIT0015] Curran A Chang IF Chang CL 2011 Calcium-dependent protein kinases from Arabidopsis show substrate specificity differences in an analysis of 103 substrates. Frontiers in Plant Science 2, 36 22645532 10.3389/fpls.2011.00036 PMC3355778

[CIT0016] Dammann C Ichida A Hong B Romanowsky SM Hrabak EM Harmon AC Pickard BG Harper JF 2003 Subcellular targeting of nine calcium-dependent protein kinase isoforms from Arabidopsis. Plant Physiology 132, 1840–1848 12913141 10.1104/pp.103.020008 PMC181270

[CIT0017] Depta H Rubery PH 1984 A comparative study of carrier participation in the transport of 2,3,5-triiodobenzoic acid, indole-3-acetic-acid, and 2,4-dichlorophenoxyacetic acid by *Cucurbita pepo* L. hypocotyl segments. Journal of Plant Physiology 115, 371–387 23194793 10.1016/S0176-1617(84)80036-X

[CIT0018] de Silva K Laska B Brown C Sederoff HW Khodakovskaya M 2011 Arabidopsis thaliana calcium-dependent lipid-binding protein (AtCLB): a novel repressor of abiotic stress response. Journal of Experimental Botany 62, 2679–2689 21252258 10.1093/jxb/erq468

[CIT0019] Dieck CB Boss WF Perera IY 2012 A role for phosphoinositides in regulating plant nuclear functions. Frontiers in Plant Science 3, 50 22645589 10.3389/fpls.2012.00050 PMC3355785

[CIT0020] Dodd AN Kudla J Sanders D 2010 The language of calcium signaling. Annual Review of Plant Biology 61, 593–620 10.1146/annurev-arplant-070109-104628 20192754

[CIT0021] Dong H Zhen ZQ Peng JY Chang L Gong QQ Wang NN 2011 Loss of ACS7 confers abiotic stress tolerance by modulating ABA sensitivity and accumulation in Arabidopsis. Journal of Experimental Botany 62, 4875–4887 21765163 10.1093/jxb/err143 PMC3193000

[CIT0022] Fasano JM Swanson SJ Blancaflor EB Dowd PE Kao TH Gilroy S 2001 Changes in root cap pH are required for the gravity response of the Arabidopsis root. The Plant Cell 13, 907–921 11283344 10.1105/tpc.13.4.907 PMC135544

[CIT0023] Felix G Grosskopf DG Regenass M Basse CW Boller T 1991 Elicitor-induced ethylene biosynthesis in tomato cells—characterization and use as a bioassay for elicitor action. Plant Physiology 97, 19–25 16668369 10.1104/pp.97.1.19 PMC1080958

[CIT0024] Ferrari S Piconese S Tronelli G Migliaccio F 2000 A new *Arabidopsis thaliana* root gravitropism and chirality mutant. Plant Science 125, 990–1000 10.1016/s0168-9452(00)00309-5 10996247

[CIT0025] Fortunati A Piconese S Tassone P Ferrari S Migliaccio F 2008 A new mutant of Arabidopsis disturbed in its roots, right-handed slanting, and gravitropism defines a gene that encodes a heat-shock factor. Journal Experimental Botany 59, 1363–1374 10.1093/jxb/ern047 18381353

[CIT0026] Friedman H Meir S Rosenberger I Halevy AH Kaufman PB Philosoph-Hadas S 1998 Inhibition of the gravitropic response of snapdragon spikes by the calcium-channel blocker lanthanum chloride. Plant Physiology 118, 483–492 9765533 10.1104/pp.118.2.483 PMC34823

[CIT0027] Friml J Vieten A Sauer M Weijers D Schwarz H Hamann T Offringa R Jurgens G 2003 Efflux-dependent auxin gradients establish the apical–basal axis of Arabidopsis. Nature 426, 147–153 14614497 10.1038/nature02085

[CIT0028] Grosskopf DG Felix G Boller T 1990 K-252a inhibits the response of tomato cells to fungal elicitors *in vivo* and their microsomal protein kinase *in vitro* . FEBS Letters 275, 177–180 2261987 10.1016/0014-5793(90)81466-2

[CIT0029] Guzman P Ecker JR 1990 Exploiting the triple response of Arabidopsis to identify ethylene-related mutants. The Plant Cell 2, 513–523 2152173 10.1105/tpc.2.6.513 PMC159907

[CIT0030] Harper JF Harmon A 2005 Plants, symbiosis and parasites: a calcium signaling connection. Nature Reviews Molecular Cell Biology 6, 555–566 10.1038/nrm1679 16072038

[CIT0031] Hasenstein KH Evans ML 1988 Effects of cations on hormone transport in primary roots of *Zea mays* . Plant Physiology 86, 890–894 11538240 10.1104/pp.86.3.890 PMC1054589

[CIT0032] Hegeman AD Rodriguez M Han BW Uno Y Phillips GN Jr Hrabak EM Cushman JC Harper JF Harmon AC Sussman MR 2006 A phyloproteomic characterization of *in vitro* autophosphorylation in calcium-dependent protein kinases. Proteomics 6, 3649–3664 16758442 10.1002/pmic.200500926

[CIT0033] Hetherington AM Trewavas A 1984 Activation of a pea membrane protein kinase by calcium ions. Planta 161, 409–417 10.1007/BF00394571 24253840

[CIT0034] Hoson T Kamisaka S Masuda Y 1996 Suppression of gravitropic response of primary roots by submergence. Planta 199, 100–104 11540722 10.1007/BF00196886

[CIT0035] Huang JZ Huber SC 2001 Phosphorylation of synthetic peptides by a CDPK and plant SNF1-related protein kinase. Influence of proline and basic amino acid residues at selected positions. Plant and Cell Physiology 42, 1079–1087 11673623 10.1093/pcp/pce137

[CIT0036] Jaspert N Throm C Oecking C 2011 Arabidopsis 14-3-3 proteins: fascinating and less fascinating aspects. Frontiers of Plant Science 2, 96 10.3389/fpls.2011.00096 PMC3355631 22639620

[CIT0037] Johannes E Collings DA Rink JC Allen NS 2001 Cytoplasmic pH dynamics in maize pulvinal cells induced by gravity vector changes. Plant Physiology 127, 119–130 11553740 10.1104/pp.127.1.119 PMC117968

[CIT0038] Kamiyoshihara Y Iwata M Fukaya T Tatsuki M Mori H 2010 Turnover of LeACS2, a wound-inducible 1-aminocyclopropane-1-carboxylic acid synthase in tomato, is regulated by phosphorylation/dephosphorylation. The Plant Journal 64, 140–150 20659278 10.1111/j.1365-313X.2010.04316.x

[CIT0039] Kieber JJ Rothenberg M Roman G Feldmann KA Ecker JR 1993 CTR1, a negative regulator of the ethylene response pathway in Arabidopsis, encodes a member of the raf family of protein kinases. Cell 72, 427–441 8431946 10.1016/0092-8674(93)90119-b

[CIT0040] Kimbrough JM Salinas-Mondragon R Boss WF Brown CS Sederoff HW 2004 The fast and transient transcriptional network of gravity and mechanical stimulation in the Arabidopsis root apex. Plant Physiology 136, 2790–2805 15347791 10.1104/pp.104.044594 PMC523342

[CIT0041] Laemmli GK 1970 Cleavage of structural proteins during the assembly of the head of bacteriophage T4. Nature 227, 680–685 5432063 10.1038/227680a0

[CIT0042] Lee LY Wu FH Hsu CT 2012 Screening a cDNA library for protein–protein interactions directly in planta. The Plant Cell 24, 1746–1759 22623495 10.1105/tpc.112.097998 PMC3442567

[CIT0043] Li N Huxtable S Yang SF Kung SD 1996 Effects of N-terminal deletions on 1-aminocyclopropane-1-carboxylate synthase activity. FEBS Letters 378, 286–290 8557119 10.1016/0014-5793(95)01464-0

[CIT0044] Li G Meng X Wang R Mao G Han L Liu Y Zhang S 2012 Dual-level regulation of ACC synthase activity by MPK3/MPK6 cascade and its downstream WRKY transcription factor during ethylene induction in Arabidopsis. PLoS Genetics 8, e1002767 22761583 10.1371/journal.pgen.1002767 PMC3386168

[CIT0045] Liang XW Abel S Keller JA Shen NF Theologis A 1992 The 1-aminocyclopropane-1-carboxylate synthase gene family of *Arabidopsis thaliana* . Proceedings of the National Academy of Sciences, USA 89, 11046–11050 10.1073/pnas.89.22.11046 PMC50480 1438312

[CIT0046] Liang X Oono Y Shen NF Köhler C Li K Scolnik PA Theologis A 1995 Characterization of two members (ACS1 and ACS3) of the 1-aminocyclopropane-1-carboxylate synthase gene family of Arabidopsis thaliana. Gene 167, 17–24 8566772 10.1016/0378-1119(95)00694-x

[CIT0047] Liese A Romeis T 2012 Biochemical regulation of *in vivo* function of plant calcium-dependent protein kinases (CDPK). Biochimica et Biophysica Acta 1833, 1582–1589 23123193 10.1016/j.bbamcr.2012.10.024

[CIT0048] Lin ZF Zhong SL Grierson D 2009 Recent advances in ethylene research. Journal of Experimental Botany 60, 3311–3336 19567479 10.1093/jxb/erp204

[CIT0049] Liu YD Zhang SQ 2004 Phosphorylation of 1-aminocyclopropane-1-carboxylic acid synthase by MPK6, a stress-responsive mitogen-activated protein kinase, induces ethylene biosynthesis in Arabidopsis. The Plant Cell 16, 3386–3399 15539472 10.1105/tpc.104.026609 PMC535880

[CIT0050] Ludwig AA Saitoh H Felix G Freymark G Miersch O Wasternack C Boller T Jones JDG Romeis T 2005 Ethylene-mediated cross-talk between calcium-dependent protein kinase and MAPK signaling controls stress responses in plants. Proceedings of the National Academy of Sciences, USA 102, 10736–10741 10.1073/pnas.0502954102 PMC1176231 16027369

[CIT0051] Luschnig C Gaxiola RA Grisafi P Fink GR 1998 EIR1, a root-specific protein involved in auxin transport, is required for gravitropism in *Arabidopsis thaliana* . Genes and Development 12, 2175–2187 9679062 10.1101/gad.12.14.2175 PMC317016

[CIT0052] Lyzenga WJ Booth JK Stone SL 2012 The Arabidopsis RING-type E3 ligase XBAT32 mediates the proteasomal degradation of the ethylene biosynthetic enzyme, 1-aminocyclopropane-1-carboxylate synthase 7. The Plant Journal 71, 23–34 22339729 10.1111/j.1365-313X.2012.04965.x

[CIT0053] Madlung A Behringer FJ Lomax TL 1999 Ethylene plays multiple nonprimary roles in modulating the gravitropic response in tomato. Plant Physiology 120, 897–906 10398726 10.1104/pp.120.3.897 PMC59329

[CIT0054] Matsunaga H Ueda H 2010 Stress-induced non-vesicular release of prothymosin-α initiated by an interaction with S100A13, and its blockade by caspase-3 cleavage. Cell Death and Differentiation 17, 1760–1772 20467443 10.1038/cdd.2010.52

[CIT0055] Mattsson J Sung ZR Berleth T 1999 Responses of plant vascular systems to auxin transport inhibition. Development 126, 2979–2991 10357941 10.1242/dev.126.13.2979

[CIT0056] McClellan CA Chang C 2008 The role of protein turnover in ethylene biosynthesis and response. Plant Science 175, 24–31 18650958 10.1016/j.plantsci.2008.01.004 PMC2293297

[CIT0057] Miller ND Durham Brooks TL Assadi AH Spalding EP 2010 Detection of a gravitropism phenotype in glutamate receptor-like 3.3 mutants of *Arabidopsis thaliana* using machine vision and computation. Genetics 186, 585–593 20647506 10.1534/genetics.110.118711 PMC2946860

[CIT0058] Morita MT 2010 Directional gravity sensing in gravitropism. Annual Review of Plant Biology 61, 705–720 10.1146/annurev.arplant.043008.092042 19152486

[CIT0059] Moseyko N Zhu T Chang HS Wang X Feldman LJ 2002 Transcription profiling of the early gravitropic response in Arabidopsis using high-density oligonucleotide probe microarrays. Plant Physiology 130, 720–728 12376639 10.1104/pp.009688 PMC166601

[CIT0060] Oliva M Dunand C 2007 Waving and skewing: how gravity and the surface of growth media affect root development in Arabidopsis. New Phytologist 176, 37–43 17692076 10.1111/j.1469-8137.2007.02184.x

[CIT0061] Peng HP Lin TY Wang NN Shih MC 2005 Differential expression of genes encoding 1-aminocyclopropane-1-carboxylate synthase in Arabidopsis during hypoxia. Plant Molecular Biology 58, 15–25 16028113 10.1007/s11103-005-3573-4

[CIT0062] Perbal G Driss-Ecole D 2003 Mechanotransduction in gravisensing cells. Trends in Plant Science 8, 498–504 14557047 10.1016/j.tplants.2003.09.005

[CIT0063] Perera IY Heilmann I Boss WF 1999 Transient and sustained increases in inositol 1,4,5-trisphosphate precede the differential growth response in gravistimulated maize pulvini. Proceedings of the National Academy of Sciences, USA 96, 5838–5843 10.1073/pnas.96.10.5838 PMC21947 10318971

[CIT0064] Perera IY Heilmann I Chang SC Boss WF Kaufman PB 2001 A role for inositol 1,4,5-trisphosphate in gravitropic signaling and the retention of cold-perceived gravistimulation of oat shoot pulvini. Plant Physiology 125, 1499–1507 11244128 10.1104/pp.125.3.1499 PMC65627

[CIT0065] Perera IY Hung CY Brady S Muday GK Boss WF 2006 A universal role for inositol 1,4,5-trisphosphate-mediated signaling in plant gravitropism. Plant Physiology 140, 746–760 16384898 10.1104/pp.105.075119 PMC1361340

[CIT0066] Perochon A Aldon D Galaud JP Ranty B 2011 Calmodulin and calmodulin-like proteins in plant calcium signaling. Biochimie 93, 2048–2053 21798306 10.1016/j.biochi.2011.07.012

[CIT0067] Philosoph-Hadas S Meri S Meir S Rosenberger I Halevy AH 1996 Regulation of the gravitropic response and ethylene biosynthesis in gravistimulated snapdragon spikes by calcium chelators and ethylene inhibitors. Plant Physiology 110, 301–310 11536726 10.1104/pp.110.1.301 PMC157721

[CIT0068] Pickett FB Wilson AK Estelle M 1990 The aux1 mutation of Arabidopsis confers both auxin and ethylene resistance. Plant Physiology 94, 1462–1466 16667854 10.1104/pp.94.3.1462 PMC1077399

[CIT0069] Plieth C Trewavas AJ 2002 Reorientation of seedlings in the earth’s gravitational field induces cytosolic calcium transients. Plant Physiology 129, 786–796 12068119 10.1104/pp.011007 PMC161701

[CIT0070] Rashotte AM Brady SR Reed RC Ante SJ Muday GK 2000 Basipetal auxin transport is required for gravitropism in roots of Arabidopsis. Plant Physiology 122, 481–490 10677441 10.1104/pp.122.2.481 PMC58885

[CIT0071] Raz V Fluhr R 1992 Calcium requirement for ethylene-dependent responses. The Plant Cell 4, 1123–1130 12297671 10.1105/tpc.4.9.1123 PMC160202

[CIT0072] Sanders D Brownlee C Harper JF 1999 Communicating with calcium. The Plant Cell 11, 691–706 10213787 10.1105/tpc.11.4.691 PMC144209

[CIT0073] Sanders D Pelloux J Brownlee C Harper JF 2002 Calcium at the crossroads of signaling. The Plant Cell 14, Suppl, S401–S417 12045291 10.1105/tpc.002899 PMC151269

[CIT0074] Santisree P Nongmaithem S Vasuki H Sreelakshmi Y Ivanchenko MG Sharma R 2011 Tomato root penetration in soil requires a coaction between ethylene and auxin signaling. Plant Physiology 156, 1424–1438 21571667 10.1104/pp.111.177014 PMC3135914

[CIT0075] Sebastià CH Hardin SC Clouse SD Kieber JJ Huber SC 2004 Identification of a new motif for CDPK phosphorylation *in vitro* that suggests ACC synthase may be a CDPK substrate. Archives of Biochemistry and Biophysics 428, 81–91 15234272 10.1016/j.abb.2004.04.025

[CIT0076] Sedbrook J Boonsirichai K Chen R 1998 Molecular genetics of root gravitropism and waving in *Arabidopsis thaliana* . Gravitational and Space Biology Bulletin 11, 71–78 11540641

[CIT0077] Sehnke PC DeLille JM Ferl RJ 2002 Consummating signal transduction: the role of 14-3-3 proteins in the completion of signal-induced transitions in protein activity. The Plant Cell 14, S339–S354 12045287 10.1105/tpc.010430 PMC151265

[CIT0078] Sinclair W Oliver I Maher P Trewavas A 1996 The role of calmodulin in the gravitropic response of the Arabidopsis thaliana agr-3 mutant. Planta 199, 343–351 8771800 10.1007/BF00195725

[CIT0079] Skottke KR Yoon GM Kieber JJ DeLong A 2011 Protein phosphatase 2A controls ethylene biosynthesis by differentially regulating the turnover of ACC synthase isoforms. PLoS Genetics 7, e1001370 21533019 10.1371/journal.pgen.1001370 PMC3080859

[CIT0080] Spanu P Grosskopf DG Felix G Boller T 1994 The apparent turnover of 1-aminocyclopropane-1-carboxylate synthase in tomato cells is regulated by protein phosphorylation and dephosphorylation. Plant Physiology 106, 529–535 12232347 10.1104/pp.106.2.529 PMC159558

[CIT0081] Strohm AK Baldwin KL Masson PH 2012 Molecular mechanisms of root gravity sensing and signal transduction. WIREs Developmental Biology 1, 276–285 23801441 10.1002/wdev.14

[CIT0082] Sukumar P Edwards KS Rahman A Delong A Muday GK 2009 PINOID kinase regulates root gravitropism through modulation of PIN2-dependent basipetal auxin transport in Arabidopsis. Plant Physiology 150, 722–735 19363095 10.1104/pp.108.131607 PMC2689958

[CIT0083] Tanaka H Dhonukshe P Brewer PB Friml J 2006 Spatiotemporal asymmetric auxin distribution: a means to coordinate plant development. Cellular and Molecular Life Sciences 63, 2738–2754 17013565 10.1007/s00018-006-6116-5 PMC11136431

[CIT0084] Tatsuki M Mori H 2001 Phosphorylation of tomato 1-aminocyclopropane-1-carboxylic acid synthase, LE-ACS2, at the C-terminal region. Journal of Biological Chemistry 276, 28051–28057 11375393 10.1074/jbc.M101543200

[CIT0085] Thomson KS Hertel R Muller S Tavares JE 1973 1-*N*-naphthylphthalamic acid and 2,3,5-triiodobenzoic acid—*in vitro* binding to particulate cell fractions and action on auxin transport in corn coleoptiles. Planta 109, 337–352 10.1007/BF00387102 24474210

[CIT0086] Toyota M Furuichi T Tatsumi H Sokabe M 2008 Critical consideration on the relationship between auxin transport and calcium transients in gravity perception of Arabidopsis seedlings. Plant Signaling and Behavior 3, 521–524 19513245 10.4161/psb.3.8.6339 PMC2634486

[CIT0087] Trewavas A 1999 Le calcium, C’est la vie: calcium makes waves. Plant Physiology 120, 1–6 10318677 10.1104/pp.120.1.1 PMC1539212

[CIT0088] Tsuchisaka A Theologis A 2004 Heterodimeric interactions among the 1-amino-cyclopropane-1-carboxylate synthase polypeptides encoded by the Arabidopsis gene family. Proceedings of the National Academy of Sciences, USA 101, 2275–2280 10.1073/pnas.0308515101 PMC356941 14983000

[CIT0089] Tsuchisaka A Yu GX Jin HL Alonso JM Ecker JR Zhang XM Gao S Theologis A 2009 A combinatorial interplay among the 1-aminocyclopropane-1-carboxylate isoforms regulates ethylene biosynthesis in *Arabidopsis thaliana* . Genetics 183, 979–1003 19752216 10.1534/genetics.109.107102 PMC2778992

[CIT0090] Utsuno K Shikanai T Yamada Y Hashimoto T 1998 Agr, an Agravitropic locus of *Arabidopsis thaliana*, encodes a novel membrane-protein family member. Plant and Cell Physiology 39, 1111–1118 9871369 10.1093/oxfordjournals.pcp.a029310

[CIT0091] Vandenbussche F Smalle J Le J 2003 The Arabidopsis mutant alh1 illustrates a cross talk between ethylene and auxin. Plant Physiology 131, 1228–1238 12644673 10.1104/pp.010850 PMC166883

[CIT0092] Wang KL Li H Ecker JR 2002 Ethylene biosynthesis and signaling networks. The Plant Cell 14, Supplement, S131–S151 12045274 10.1105/tpc.001768 PMC151252

[CIT0093] Wang KL Yoshida H Lurin C Ecker JR 2004 Regulation of ethylene gas biosynthesis by the Arabidopsis ETO1 protein. Nature 428, 945–950 15118728 10.1038/nature02516

[CIT0094] Went FA 1928 The international union of biological sciences. Science 68, 545–547 17736406 10.1126/science.68.1770.545-a

[CIT0095] Wolverton C Paya AM Toska J 2011 Root cap angle and gravitropic response rate are uncoupled in the Arabidopsis *pgm-1* mutant. Physiologia Plantarum 141, 373–382 21143486 10.1111/j.1399-3054.2010.01439.x

[CIT0096] Wu K Lu GH Sehnke P Ferl RJ 1997 The heterologous interactions among plant 14-3-3 proteins and identification of regions that are important for dimerization. Archives of Biochemistry and Biophysics 339, 2–8 9056226 10.1006/abbi.1996.9841

[CIT0097] Yamagami T Tsuchisaka A Yamada K Haddon WF Harden LA Theologis A 2003 Biochemical diversity among the 1-amino-cyclopropane-1-carboxylate synthase isozymes encoded by the Arabidopsis gene family. Journal of Biological Chemistry 278, 49102–49112 12968022 10.1074/jbc.M308297200

[CIT0098] Yang SF Hoffman NE 1984 Ethylene biosynthesis and its regulation in higher plants. Annual Review of Plant Physiology 35, 155–189

[CIT0099] Yao Y Du Y Jiang L Liu JY 2007 Interaction between ACC synthase 1 and 14-3-3 proteins in rice: a new insight. Biochemistry (Moscow) 72, 1003–1007 17922660 10.1134/s000629790709012x

[CIT0100] Yip WK Dong JG Yang SF 1991 Purification and characterization of 1-aminocyclopropane-1-carboxylate synthase from apple fruits. Plant Physiology 95, 251–257 16667960 10.1104/pp.95.1.251 PMC1077514

[CIT0101] Yoo SD Cho YH Sheen J 2007 Arabidopsis mesophyll protoplasts: a versatile cell system for transient gene expression analysis. Nature Protocols 2, 1565–1572 10.1038/nprot.2007.199 17585298

[CIT0102] Yoo SD Cho Y Sheen J 2009 Emerging connections in the ethylene signaling network. Trends in Plant Science 14, 270–279 19375376 10.1016/j.tplants.2009.02.007 PMC3063992

[CIT0103] Yoon GM Kieber JJ 2013 14-3-3 regulates 1-aminocyclopropane-1-carboxylate synthase protein turnover in Arabidopsis. The Plant Cell 25, 1016–1028 23512855 10.1105/tpc.113.110106 PMC3634674

[CIT0104] Yoshida H Nagata M Saito K Wang KL Ecker JR 2005 Arabidopsis ETO1 specifically interacts with and negatively regulates type 2 1-aminocyclopropane-1-carboxylate synthases. BMC Plant Biology 5, 14 16091151 10.1186/1471-2229-5-14 PMC1199607

[CIT0105] Yoshida H Wang LC Chang CM Mori K Uchida E Ecker JR 2006 The ACC synthase TOE sequence is required for interaction with ETO1 family proteins and destabilization of target proteins. Plant Molecular Biology 62, 427–437 16897471 10.1007/s11103-006-9029-7

[CIT0106] Yuan S Dean JF 2010 Differential responses of the promoters from nearly identical paralogs of loblolly pine (*Pinus taeda L.*) ACC oxidase to biotic and abiotic stresses in transgenic *Arabidopsis thaliana* . Planta 232, 873–886 20632186 10.1007/s00425-010-1224-8

