# Supplementary material for: A type III ACC synthase, ACS7, is involved in root gravitropism in Arabidopsis thaliana
Source: J Exp Bot. 2013 Aug 13;64(14):4343–60. doi: 10.1093/jxb/ert241 (PMC3808318; doi:10.1093/jxb/ert241)
Supplement: Supplementary Data [file supp_64_14_4343__index.html]

A type III ACC synthase, ACS7, is involved in root gravitropism in Arabidopsis thaliana — A type III ACC synthase, ACS7, is involved in root gravitropism in Arabidopsis thaliana — Supplementary Data 

# A type III ACC synthase, *ACS7*, is involved in root gravitropism in *Arabidopsis thaliana*

## 

Data files

**Files in this Data Supplement:**

- Supplementary Data - Supplementary Data
