# Supplementary material for: A type III ACC synthase, ACS7, is involved in root gravitropism in Arabidopsis thaliana
Source: J Exp Bot. 2013 Aug 13;64(14):4343–60. doi: 10.1093/jxb/ert241 (PMC3808318; doi:10.1093/jxb/ert241)

**Supplementary Table S1**

Information of fusion peptides.

| Name | Amino acid sequence |
| --- | --- |
| Di19-2-2 WT | DVLKSEQKEMpSYREDPY |
| Di19-2-2 MT | DVLKSEQKEMAYREDPY |
| NR | TLKRTApSTPFM |
| LeACS2 | KKNNLRLpSFSKRMY |
| ACS5 P1-1 | KSLRKKTVSNWVF |
| ACS7-P1-1 | RVAVSDTHGED |
| ACS7-P2-1 | NPEGSMWG |
| ACS7-P3-3 | FRQAMASFMEQIR |
| ACS7-P4-5 | VLITNPSNPL |
| ACS7-P5-2 | VGTIYSYNDNV |
| ACS7-P4T-2-2 | SNPLGATVQKKVL |
| ACS7-P5T-3 | PGFRVGTIYSYND |
| ACS7-P6-2 | TARRMSSFTLVSS |
| ACS7-P7-1 | NNNVELSRVAVSD |
| ACS7-P8-1 | DTHGEDSPYFAGW |
| ACS7-P9-3 | EGSMWGSKGAPGF |
| ACS7-P10-1 | DYHGLKTFRQAMA |
| ACS7-P11-2 | VRGVLITNPSNPL |

| | |
|---|---|
| ACS7-P5-2 S→A | VGTIYAYNDNV |
| ACS7-P5-2 T→A | VGAIYSYNDNV |
| ACS7-P5-2 TS→AA | VGAIYAYNDNV |
| ACS7-P11-2 S→A | VRGVLITNPANPL |
| ACS7-P11-2 T→A | VRGVLIANPSNPL |
| ACS7-P11-2 TS→AA | VRGVLIANPANPL |
| ACS7-P4-5 S→A | VLITNPANPL |
| ACS7-P4-5 T→A | VLIANPSNPL |
| ACS7-P4-5 TS→AA | VLIANPANPL |
| ACS7-P5T-3 S→A | PGFRVGTIYAYND |
| ACS7-P5T-3 T→A | PGFRVGAIYSYND |
| ACS7-P5T-3 TS→AA | PGFRVGAIYAYND |

A. pS represents phosphor-serine

B. Gray bars represent point mutated amino acid residue

**Supplementary Fig. S1**

Plasmid maps

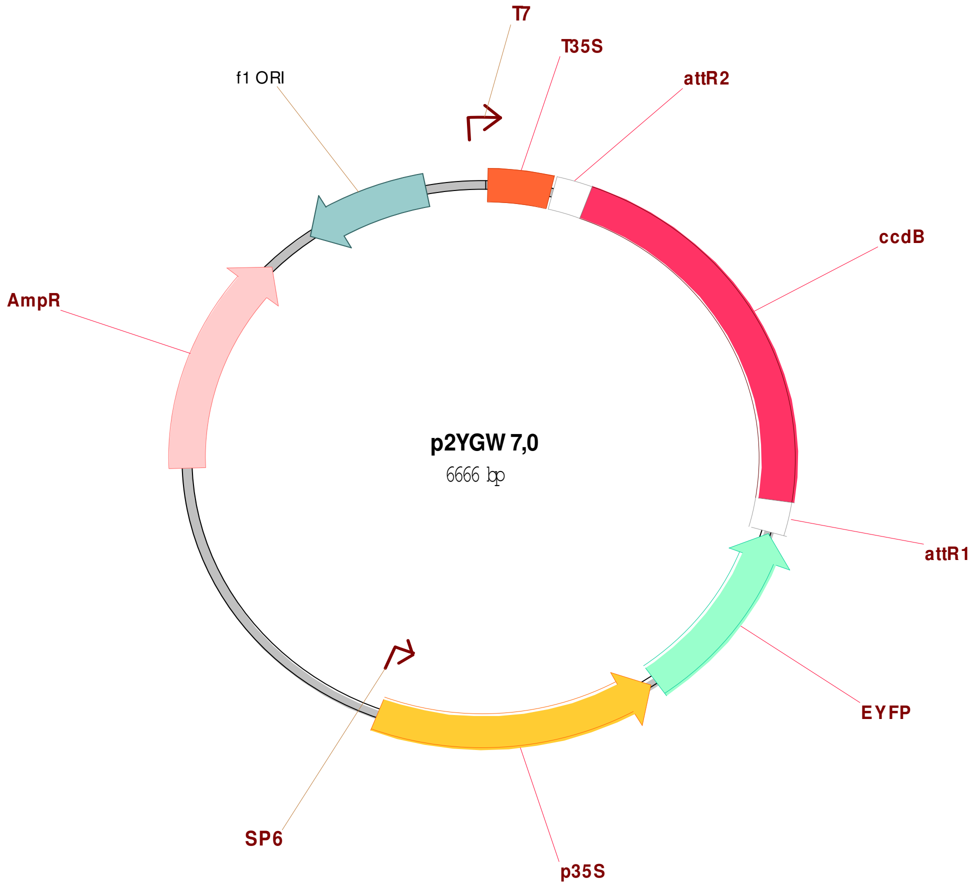


**Plasmid map of p2YGW7 which is used to determine the localization of ACS7 in protoplast**

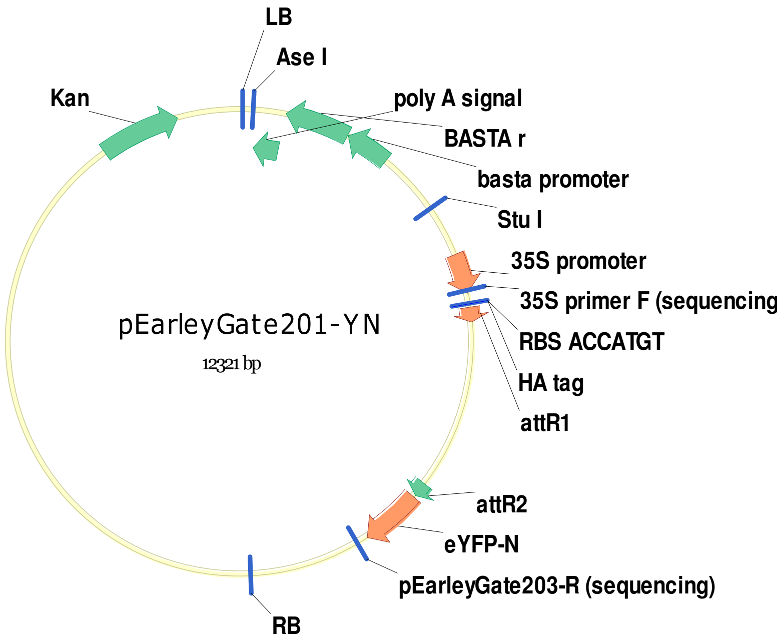


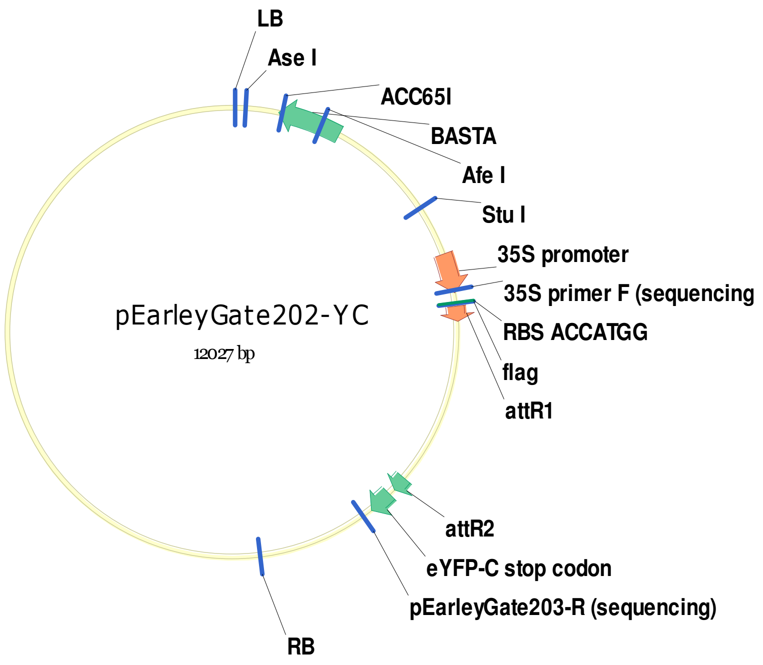


**Plasmid map of pEarleyGate201-YN and pEarleyGate201-YC that are used for BiFC assay**

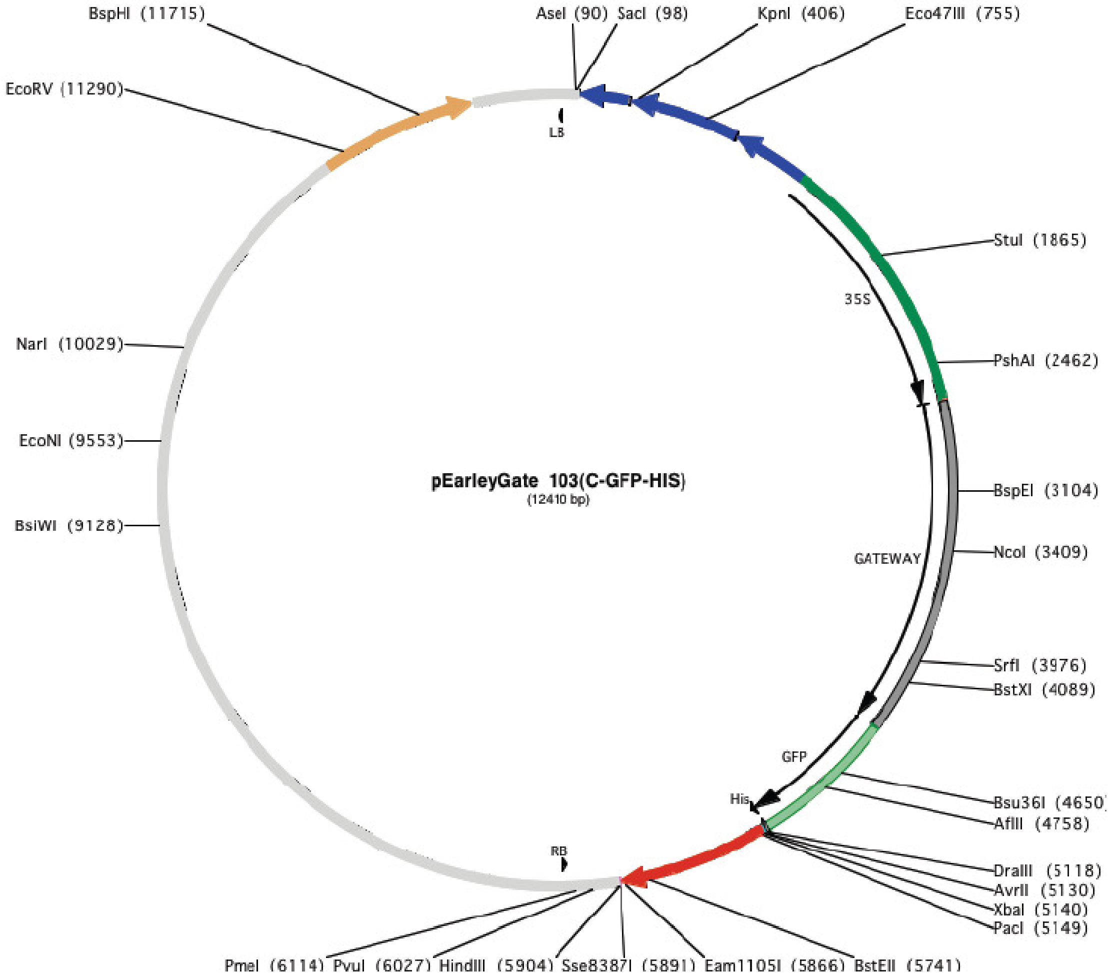


**Plasmid map of pEarleyGate103 which is used to produce ACS7 overexpression line and complementation of *acs7-1* mutant line**

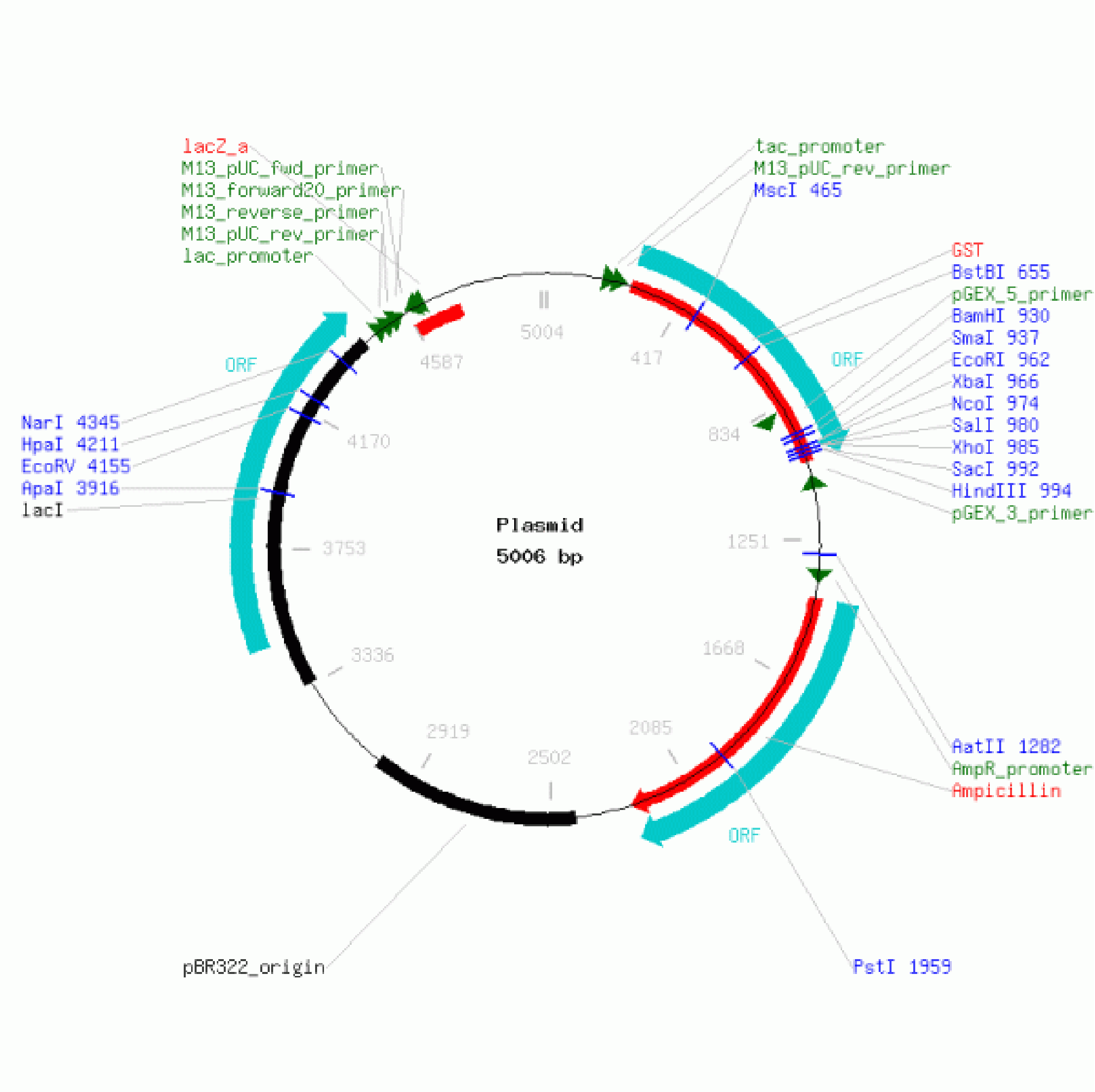


**Plasmid map of NRV empty vector which is used for all fusion peptide constructs**

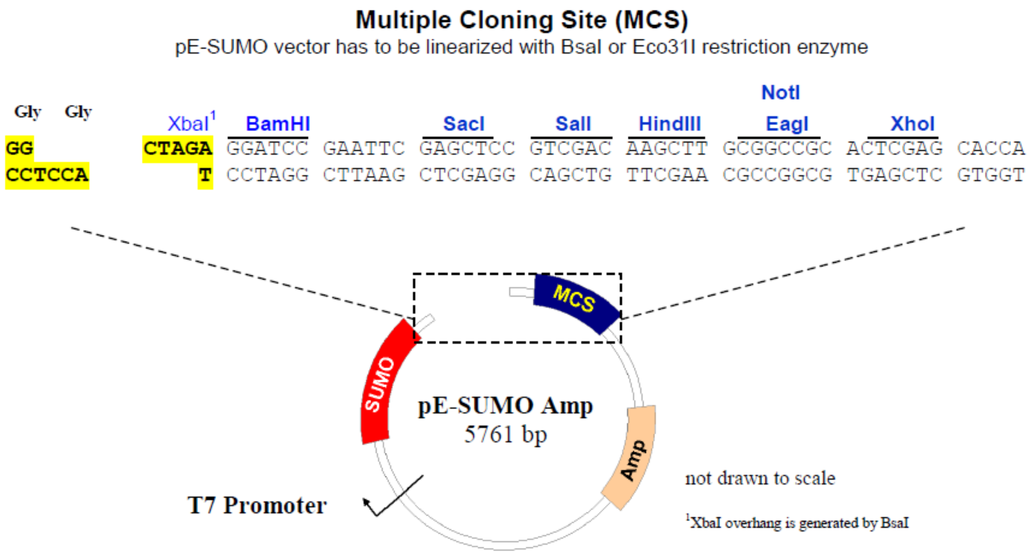


**Plasmid of pE-SUMO which is used to produce 6His-SUMO-ACS7 for enzyme activity assay**

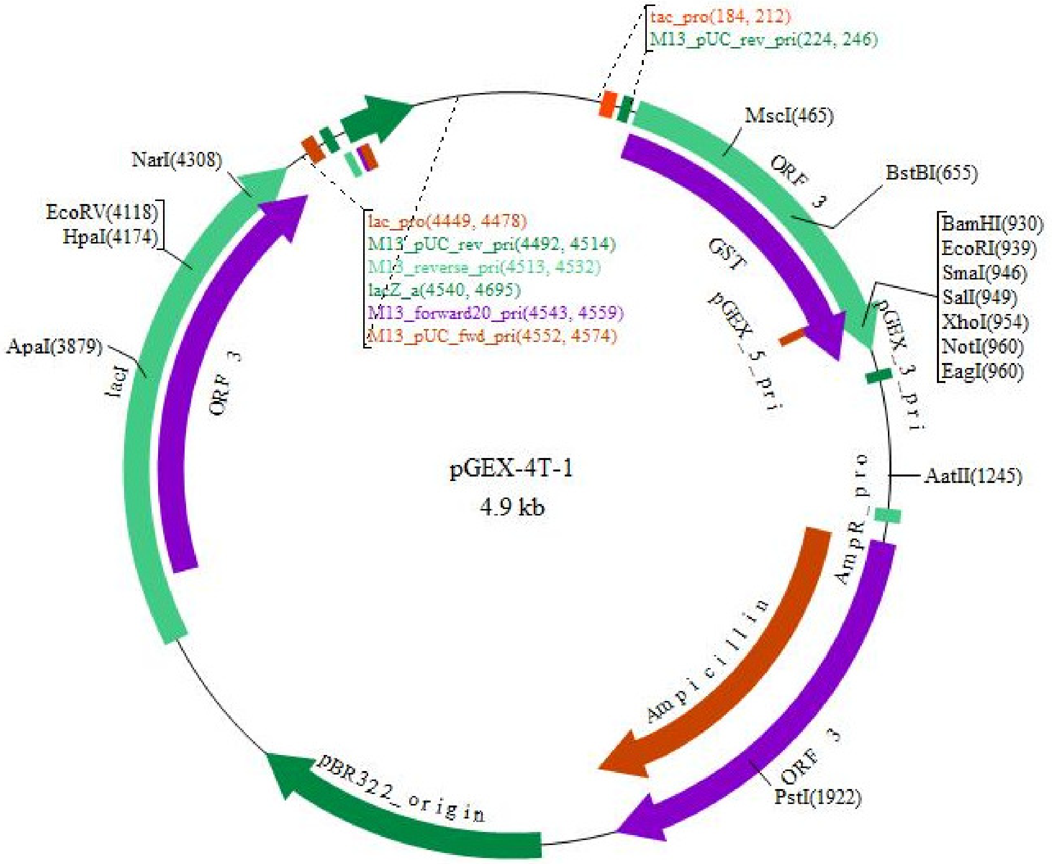


**Plasmid of pGEX-4T-1**

**Supplementary Fig. S2.** Three-day-old seedlings were grown on the agar plate for 12 to 24 hours followed by 90 degrees counterclockwise reorientation. Root curvature and percentage of gravity sustaining (gravity response) was measured every 12 hours.

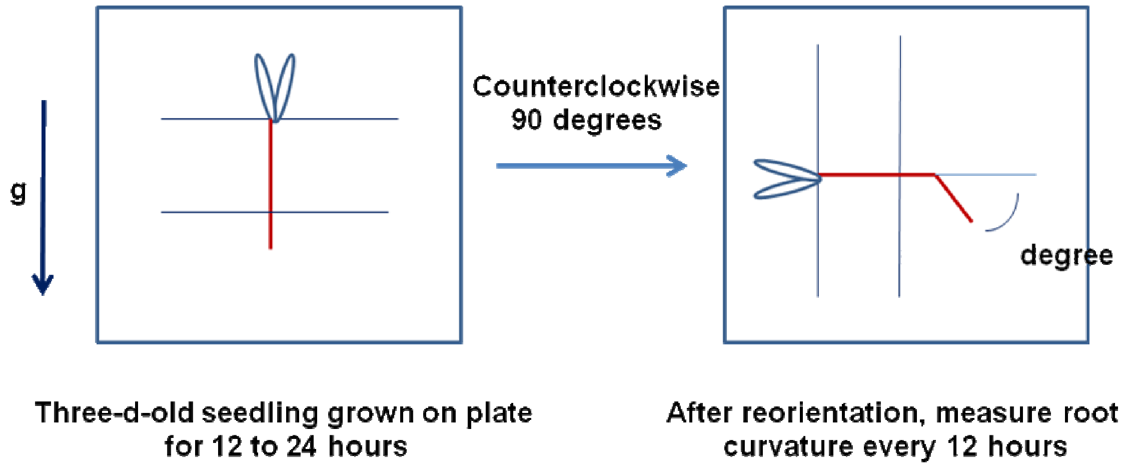

**Supplementary Fig. S3.** Bar graph of the phosphorylation signal in the *in vitro* kinase assay using mutated AtCDPK16. The mutated AtCDPK16 showed reduced autophosphorylation. The autophosphorylation signals of wild type (G-CDPK16-6His) and mutant kinase (G-CDPK16-6His MT) and the phosphorylation signals of AtACS7 fusion peptides were normalized to the coomassie blue stained protein intensity. The results showed that when mutated AtCDPK16 was used, the phosphorylation signals of AtACS7 fusion peptides decreased. This indicates that the phosphorylation of AtACS7 fusion peptides was resulted from AtCDPK16 but not from other bacterial kinases.

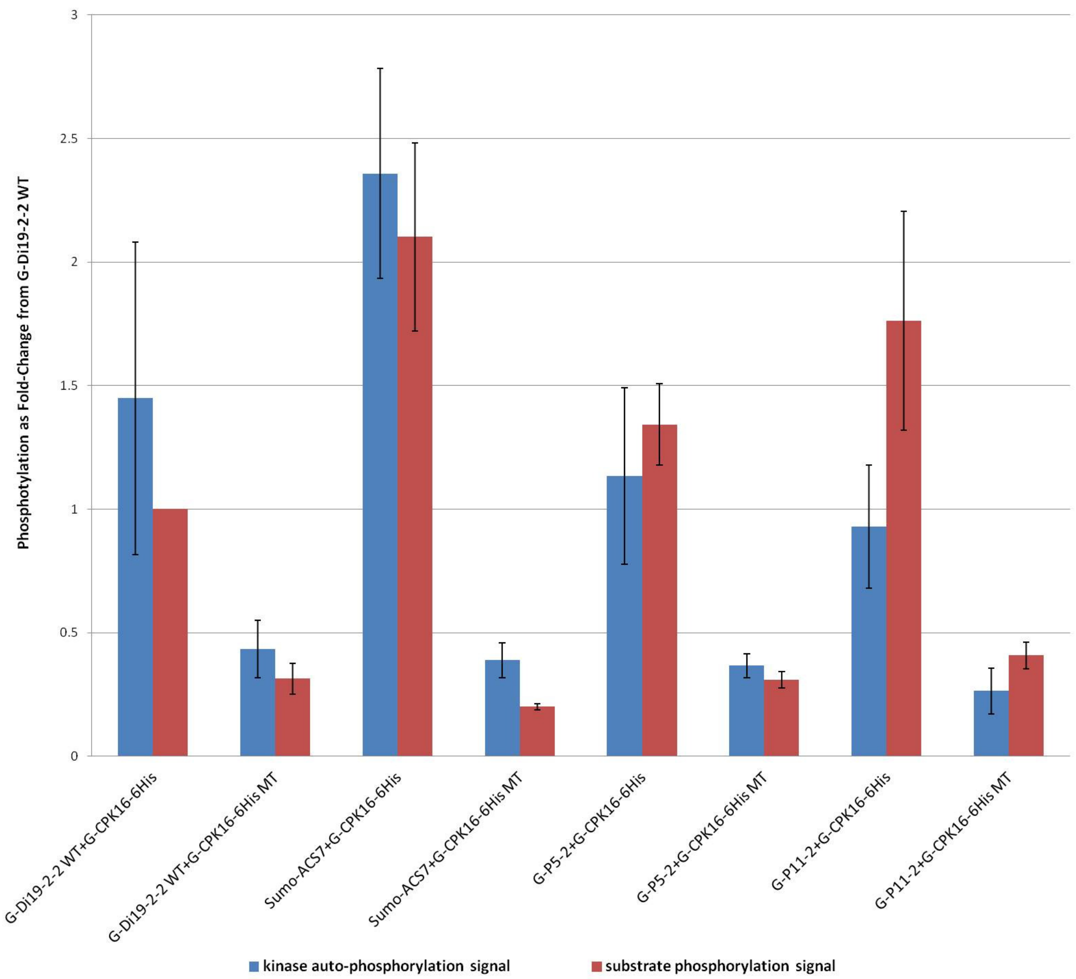

**Supplementary Fig. S4.** MS/MS fragmentation pattern of a phosphorylated peptide (VGTIYSYNDNVVR) of ACS7 recombinant protein. The fragmentation pattern was validated using Scaffold software (version 2.0). The spectrum indicated that Ser-299 is phosphorylated.

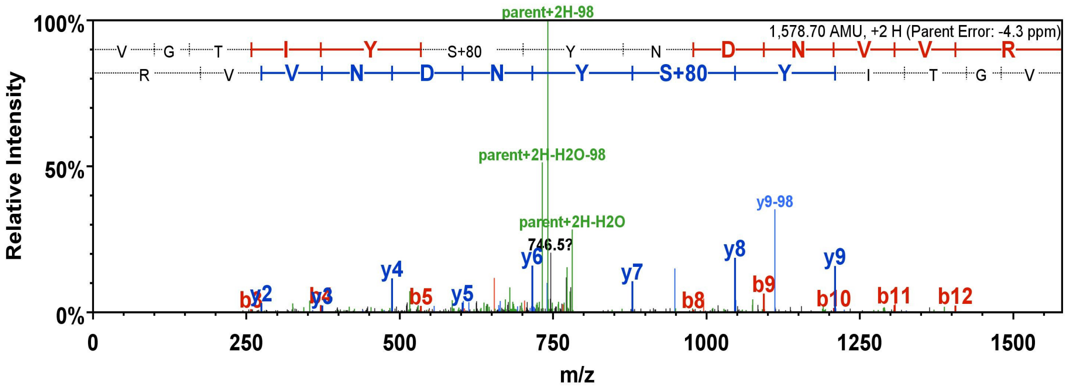


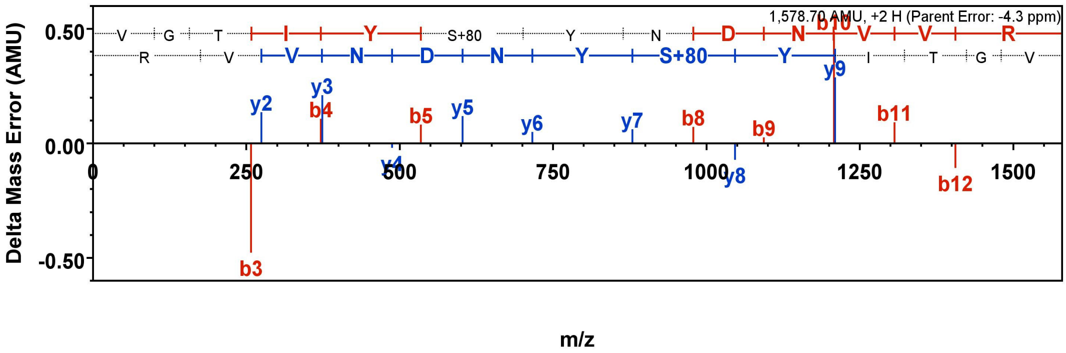


| B | B Ions | B+2H | B-NH3 | B-H2O | AA | Y Ions | Y+2H | Y-NH3 | Y-H2O | Y |
|---|---|---|---|---|---|---|---|---|---|---|
| 1 | 100.1 | | | | V | 1,579.7 | 790.4 | 1,562.7 | 1,561.7 | 13 |
| 2 | 157.1 | | | | G | 1,480.6 | 740.8 | 1,463.6 | 1,462.6 | 12 |
| 3 | 258.1 | | | 240.1 | T | 1,423.6 | 712.3 | 1,406.6 | 1,405.6 | 11 |
| 4 | 371.2 | | | 353.2 | I | 1,322.6 | 661.8 | 1,305.6 | 1,304.6 | 10 |
| 5 | 534.3 | | | 516.3 | Y | 1,209.5 | 605.3 | 1,192.5 | 1,191.5 | 9 |
| 6 | 701.3 | 351.1 | | 683.3 | S+80 | 1,046.4 | 523.7 | 1,029.4 | 1,028.4 | 8 |
| 7 | 864.4 | 432.7 | | 846.3 | Y | 879.4 | 440.2 | 862.4 | 861.4 | 7 |
| 8 | 978.4 | 489.7 | 961.4 | 960.4 | N | 716.4 | 358.7 | 699.3 | 698.4 | 6 |
| 9 | 1,093.4 | 547.2 | 1,076.4 | 1,075.4 | D | 602.3 | | 585.3 | 584.3 | 5 |
| 10 | 1,207.5 | 604.2 | 1,190.4 | 1,189.5 | N | 487.3 | | 470.3 | | 4 |
| 11 | 1,306.5 | 653.8 | 1,289.5 | 1,288.5 | V | 373.3 | | 356.2 | | 3 |
| 12 | 1,405.6 | 703.3 | 1,388.6 | 1,387.6 | V | 274.2 | | 257.2 | | 2 |
| 13 | 1,579.7 | 790.4 | 1,562.7 | 1,561.7 | R | 175.1 | | 158.1 | | 1 |

**Supplementary Fig. S5.** Protein-protein interaction between ACS7 and 14-3-3ω using BiFC analysis. (A) Co-expression of cEYFP and nEYFP (negative control). (B) Co-expression of ACS7-cEYFP and ACS7-nYFP (positive control). (C) Co-expression of NR-nEYFP and 14-3-3-cEYFP (positive control). (D) Co-expression of NR-cEYFP and 14-3-3-nEYFP (positive control). (E) Co-expression of ACS7-nEYFP and 14-3-3cEYFP. (F) Co-expression of ACS7-cEYFP and 14-3-3YFP. Figure (E) and (F) are for ACS7 and 14-3-3ωinteraction. YFP signals were observed by confocal microscopy. Blue signal shows nucleus stained with DAPI; red signal showed chlorophyll with auto-fluorescent light; merge showed YFP, DAPI, chlorophyll and bright field signals.

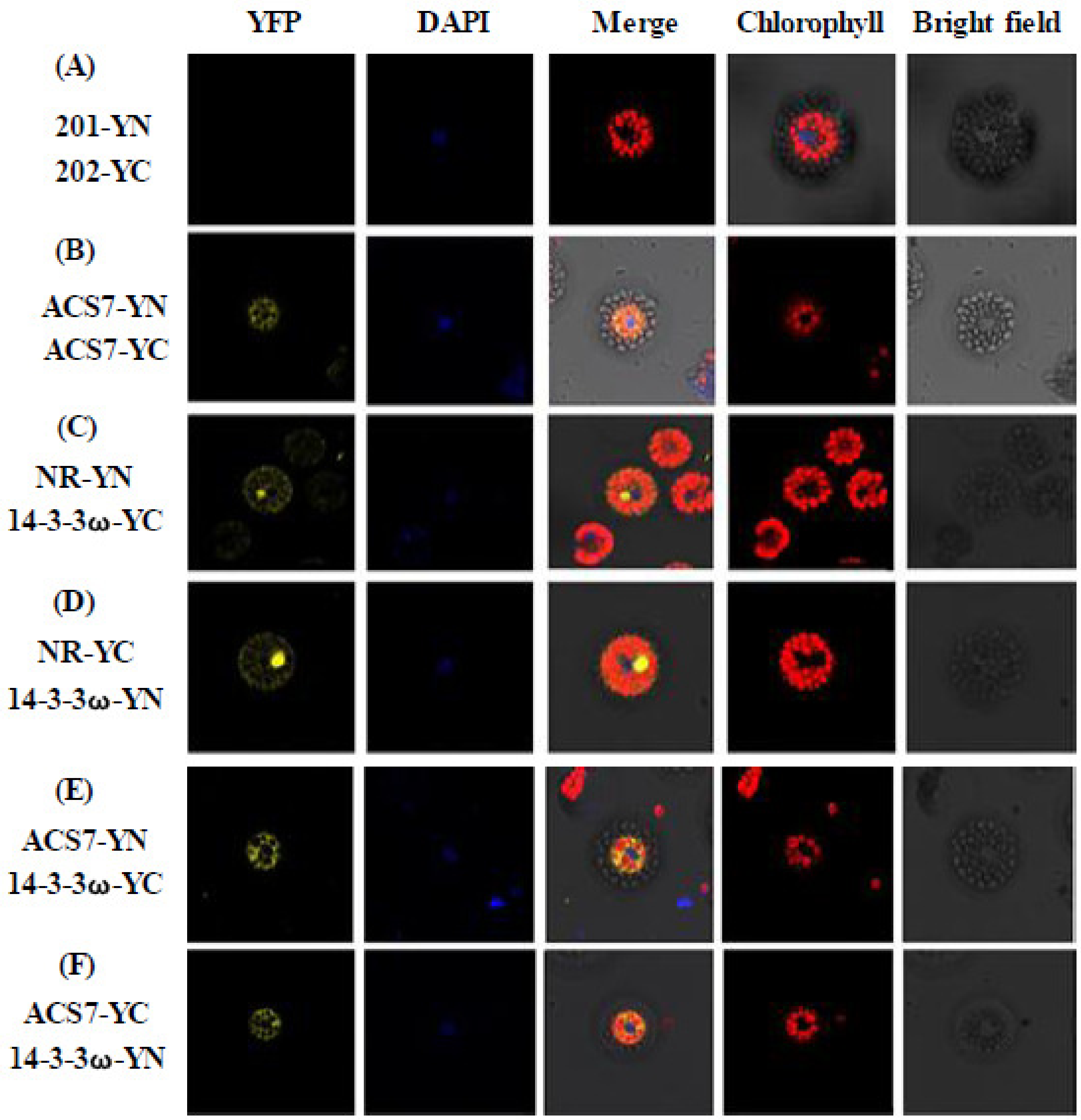

**Supplementary Fig. S6.** Quartz Crystal Microbalance (QCM) and kinetic analysis of protein-protein interaction between recombinant protein (A) 6His-SUMO-AtACS7 and G-14-3-3ω or (B) phosphorylated 6His-SUMO-AtACS7 by AtCDPK16 and G-14-3-3ω. 6His-SUMO-AtACS7 protein was phosphorylated by AtCDPK16 *in vitro* in advance. G-14-3-3ω protein was coated on the sensor and (A) 6His-SUMO-AtACS7 protein or (B) phosphorylated 6His-SUMO-AtACS7 protein was injected into the buffer. The frequency change of 6His-SUMO-AtACS7 with G-14-3-3ω (A) and phosphorylated 6His-SUMO-AtACS7 with G-14-3-3ω (B) were analyzed by AFFINIXQN software. Arrows indicated the timing of 6His-SUMO-AtACS7 or phosphorylated 6His-SUMO-AtACS7 which was injected. QCM data of protein-protein interaction between 6His-SUMO-AtACS7 and G-14-3-3ω or phosphorylated 6His-SUMO-AtACS7 and G-14-3-3ω were analyzed by AQUA software. The average Kd and SE of three independent experiments of (C) AtACS7 and G-14-3-3ω was $9.29 \pm 1.99 \times 10^{-9}$M and (D) the phosphorylated AtACS7 and G-14-3-3ω was $5.05 \pm 1.96 \times 10^{-9}$M. For control experiment (negative control), Quartz Crystal Microbalance (QCM) analysis was carried for protein-protein interaction between recombinant protein (E) G-14-3-3ω and tag only protein 6His-SUMO and (F) 6His-SUMO-ACS7 and tag only protein GST. The results indicated that the delta frequency of (A) 6His-SUMO with G-14-3-3ω protein and (B) GST with 6His-SUMO-ACS7 protein were not changed. Arrows indicate the injection of the second protein.

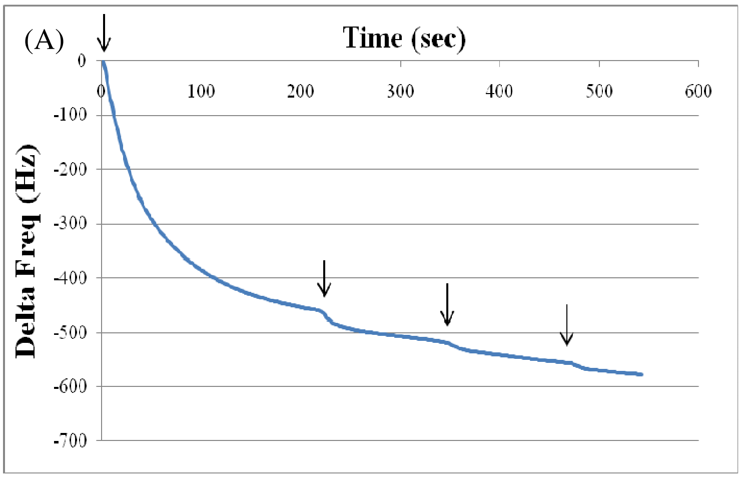

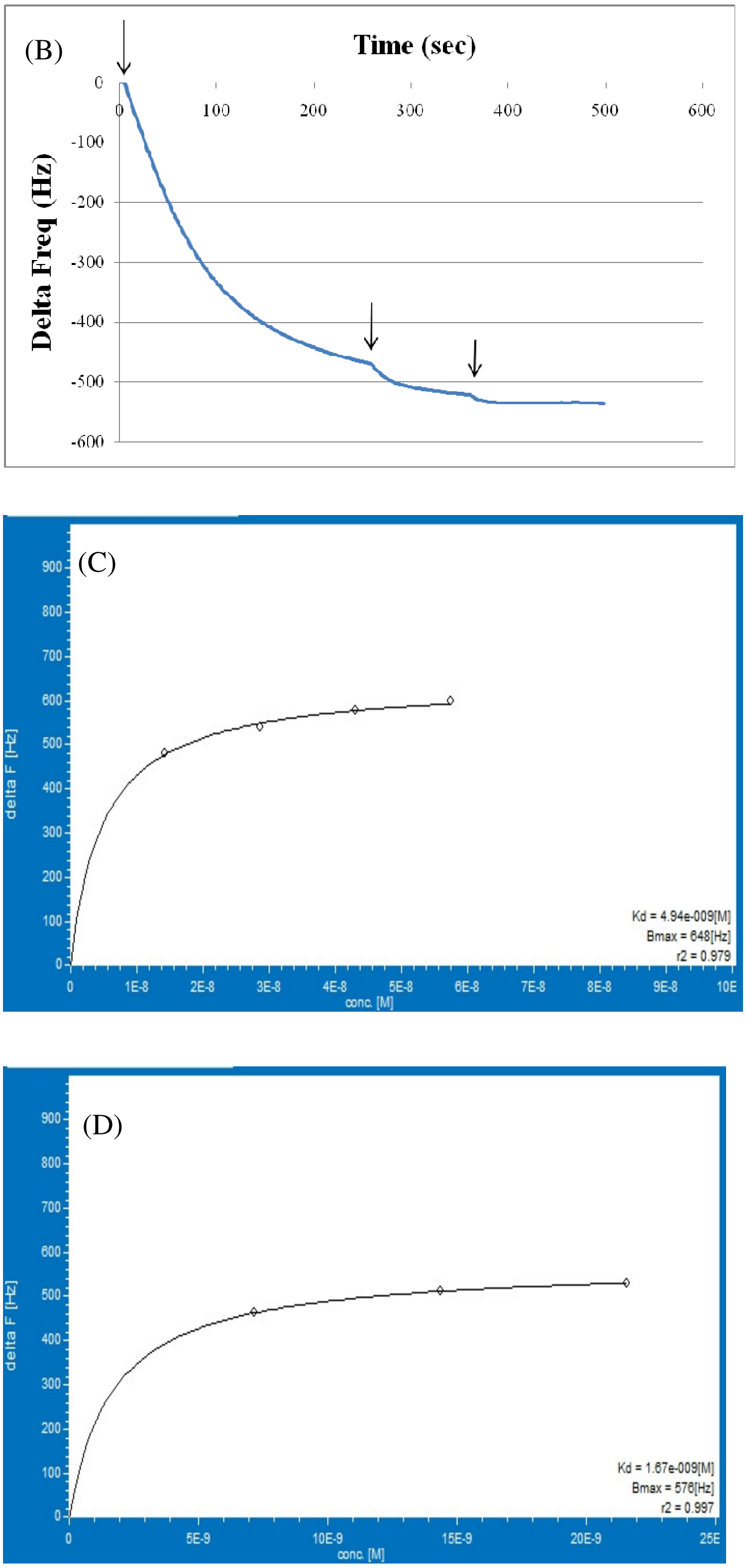
(B)
Time (sec)
Delta Freq (Hz)
0
100
200
300
400
500
600
-100
-200
-300
-400
-500
-600
(C)
delta F [Hz]
conc. [M]
Kd = 4.94e-009[M]
Bmax = 648[Hz]
r2 = 0.979
(D)
delta F [Hz]
conc. [M]
Kd = 1.67e-009[M]
Bmax = 576[Hz]
r2 = 0.997

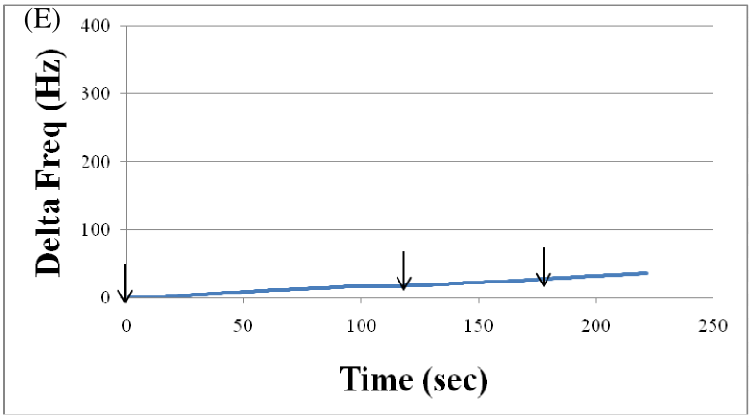
(E)
Delta Freq (Hz)
400
300
200
100
0
0
50
100
150
200
250
Time (sec)

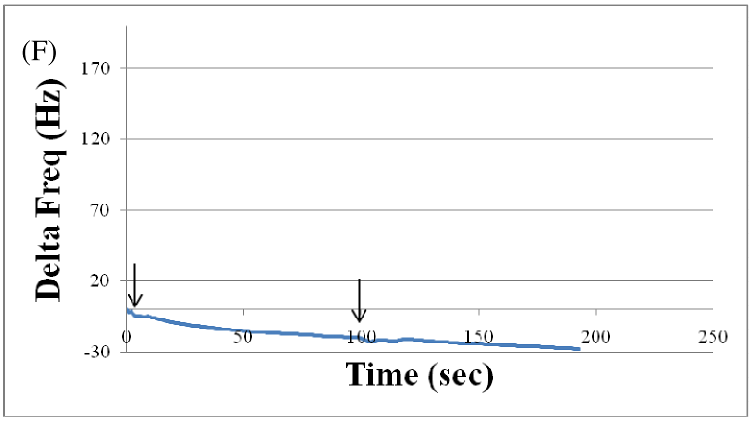
(F)
Delta Freq (Hz)
170
120
70
20
-30
0
50
100
150
200
250
Time (sec)

**Supplementary Fig. S7.** Isolation of T-DNA insertional mutant *acs7-1*. In order to investigate the ACS7 protein functions, *acs7-1* mutant was ordered from ABRC. Panel (A) is T-DNA insertion site (Exon 3) in *AtACS7-1* (CS_16570). Panel (B) is genotyping of *acs7-1* mutant by PCR. The result showed that *acs7-1* is a homozygous mutant.

(A)

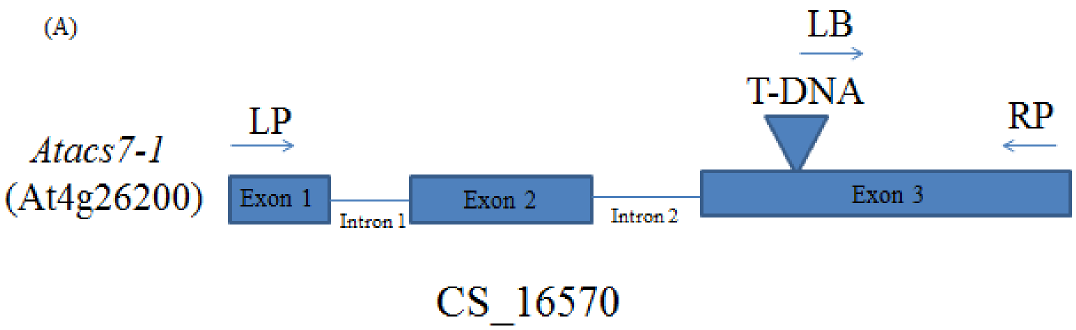


(B)

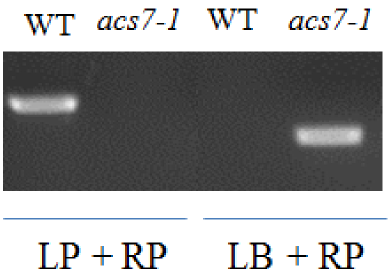

**Supplementary Fig. S8.** Gravity response of WT and *acs7-1* mutant affected by 0.5 mM $LaCl_3$. Three days old WT and *acs7-1* seedlings were transferred to the 0.5 mM $LaCl_3$ containing plate growing for one day and turned the plate left to 90 degree for two days to observe the gravity response.Gravity vector shows as arrowhead indicates after one day growth. The result showed both of WT and *acs7-1* mutant lost gravity response.

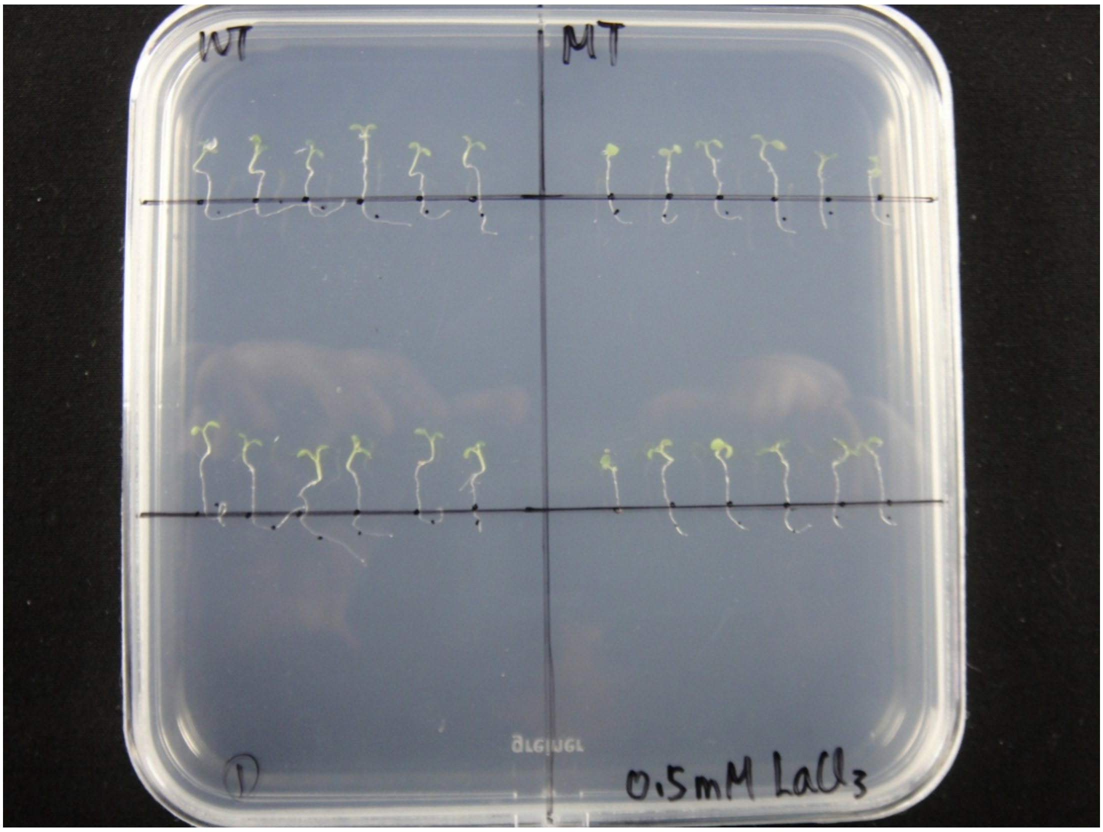

**Supplementary Fig. S9.** The effect of 50 μM CPZ on WT and *acs7-1* mutant. Three days old WT and *acs7-1* seedlings were transferred to the 50 μM CPZ containing plate growing for one day to observe the root elongation. The result showed both of WT and *acs7-1* mutant root elongation were totally inhibited.

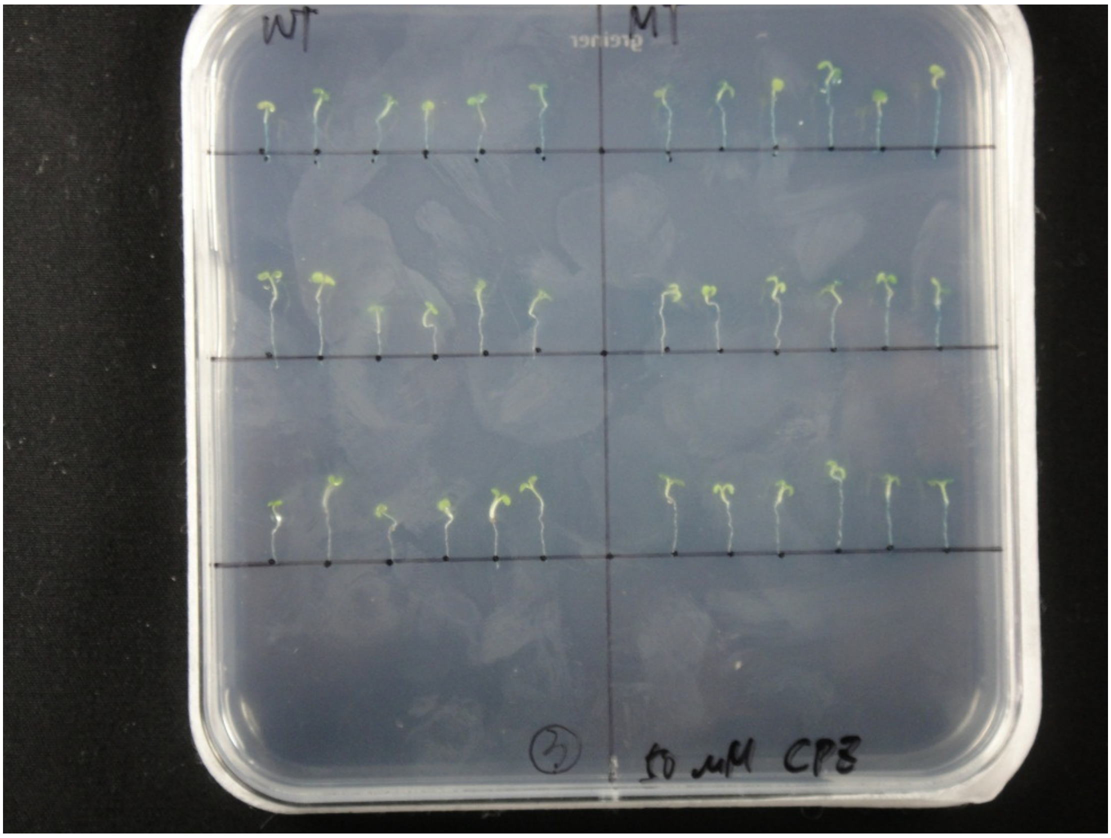

Supplement: Supplementary Data [file supp_ert241_jexbot096719_file001.pdf]
